# Endothelial Cell‐Derived Extracellular Vesicles Promote Aberrant Neutrophil Trafficking and Subsequent Remote Lung Injury

**DOI:** 10.1002/advs.202400647

**Published:** 2024-08-09

**Authors:** Shuang‐Feng Zi, Xiao‐Jing Wu, Ying Tang, Yun‐Peng Liang, Xu Liu, Lu Wang, Song‐Li Li, Chang‐De Wu, Jing‐Yuan Xu, Tao Liu, Wei Huang, Jian‐Feng Xie, Ling Liu, Jie Chao, Hai‐Bo Qiu

**Affiliations:** ^1^ Jiangsu Provincial Key Laboratory of Critical Care Medicine Department of Critical Care Medicine Zhongda Hospital School of Medicine Southeast University Nanjing 210009 China; ^2^ Department of Biochemistry and Molecular Biology School of Medicine Southeast University Nanjing 210009 China; ^3^ Department of Physiology School of Medicine Southeast University Nanjing 210009 China

**Keywords:** ALI/ARDS, endothelial cells, extracellular vesicles, neutrophils, sepsis

## Abstract

The development of acute respiratory distress syndrome (ARDS) in sepsis is associated with substantial morbidity and mortality. However, the molecular pathogenesis underlying sepsis‐induced ARDS remains elusive. Neutrophil heterogeneity and dysfunction contribute to uncontrolled inflammation in patients with ARDS. A specific subset of neutrophils undergoing reverse transendothelial migration (rTEM), which is characterized by an activated phenotype, is implicated in the systemic dissemination of inflammation. Using single‐cell RNA sequencing (scRNA‐seq), it identified functionally activated neutrophils exhibiting the rTEM phenotype in the lung of a sepsis mouse model using cecal ligation and puncture. The prevalence of neutrophils with the rTEM phenotype is elevated in the blood of patients with sepsis‐associated ARDS and is positively correlated with disease severity. Mechanically, scRNA‐seq and proteomic analys revealed that inflamed endothelial cell (EC) released extracellular vesicles (EVs) enriched in karyopherin subunit beta‐1 (KPNB1), promoting abluminal‐to‐luminal neutrophil rTEM. Additionally, EC‐derived EVs are elevated and positively correlated with the proportion of rTEM neutrophils in clinical sepsis. Collectively, EC‐derived EV is identified as a critical regulator of neutrophil rTEM, providing insights into the contribution of rTEM neutrophils to sepsis‐associated lung injury.

## Graphical abstract

It is shown that endothelial cell (EC)‐derived extracellular vesicles (EVs) promote the reverse transendothelial migration (rTEM) of PMNs with a highly activated phenotype, contributing to distant lung injury during sepsis. Mechanistically, this aberrant response is caused by degradation of junctional adhesion molecule‐C (JAM‐C) at EC junctions by neutrophil elastase (NE), partially mediated by KPNB1 contained in inflamed EC‐derived EVs.

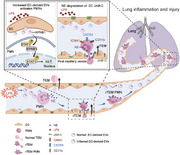

## 1 Introduction

Sepsis is a life‐threatening condition characterized by a dysregulated immune response to systemic infection, leading to multiple organ failure, secondary infections and ultimately mortality.^[^ ^1^ ^]^ The development of acute respiratory distress syndrome (ARDS) has been identified as an independent risk factor for in‐hospital mortality, contributing to up to 37% of deaths in individuals with sepsis.^[^ ^2^ ^]^ Despite improvements in critical care and organ support techniques, no effective pharmacotherapies are currently available for sepsis.^[^ ^1b^ ^]^ Therefore, gaining a comprehensive understanding of the molecular mechanisms underlying sepsis‐induced ARDS may provide crucial insights into disease pathophysiology and therapeutic opportunities.

Immune disorders play an important role in the cytokine storms and subsequent organ damage during sepsis.^[^ ^3^ ^]^ Polymorphonuclear neutrophils (PMNs) are the most abundant immune cells in circulation and are the first responders to inflammation or infection.^[^ ^4^ ^]^ Abnormal infiltration and functional overactivation of PMNs have deleterious effects on normal tissues.^[^ ^5^ ^]^ Phenotypic heterogeneity and functional diversity of PMNs have been documented under both homeostatic and pathological conditions.^[^ ^6^ ^]^ Notably, a distinct subset of PMNs can reenter the bloodstream in an abluminal‐to‐luminal direction within initially inflamed tissues. This process is known as PMN reverse transendothelial migration (PMN rTEM).^[^ ^7^ ^]^ PMNs that have undergone rTEM are referred to as rTEM PMNs. Traditionally, PMNs have been believed to undergo apoptosis and are subsequently eliminated through macrophage phagocytosis at the inflamed site.^[^ ^8^ ^]^ However, rTEM PMNs are predisposed to be programmed toward a highly activated phenotype with prolonged survival, which contribute to remote organ injury.^[^ ^9^ ^]^ While various mechanisms, such as chemoattractant sources at the wound and vasculature, and chemokine receptor desensitization,^[^ ^10^ ^]^ have been proposed to elucidate the abnormal trafficking of PMNs from inflamed tissues, the essential factors orchestrating the reverse migration of PMNs from sites of tissue damage remain largely unexplored. Given that rTEM PMNs can circulate and propagate remote inflammation via altered cellular trafficking and function, investigating their potential involvement in initiating lung injury during sepsis is imperative. Uncovering the key mechanisms governing the rTEM of PMNs in the context of sepsis is essential.

Endothelial cells (ECs) within blood vessels are recognized for their immunomodulatory capacity, which orchestrate the regulation of immune cell trafficking, activation status, and function.^[^ ^11^ ^]^ The PMN transendothelial migration cascade, also known the PMN extravasation cascade, is orchestrated by a series of intricate and sequential interactions between ECs and PMNs involving various adhesion receptors.^[^ ^12^ ^]^ Moreover, ECs serve as crucial participants in the reverse transendothelial process of PMNs under inflammatory environment. Secretion of extracellular vesicles (EVs) has emerged as a predominant mechanism that facilitates contact‐independent interactions between cells and their environment.^[^ ^13^ ^]^ EVs encapsulate a diverse array of bioactive cargo originating from the parent cells, positioning themselves as prominent regulators of immunomodulation during inflammation progression.^[^ ^14^ ^]^ In our previous study, we demonstrated a substantial increase in EVs from ECs within the bloodstream during bacterial pneumonia in ex vivo perfused human lungs.^[^ ^15^ ^]^ Emerging evidence indicates that EC‐derived EVs play a role in immune cell activation and migration.^[^ ^16^ ^]^ Nonetheless, the specific involvement of EC‐derived EVs in triggering rTEM of PMNs remains to be elucidated.

In this study, we investigated the occurrence of rTEM PMNs and their contribution to distant lung injury in sepsis. Furthermore, we elucidated the potential role of EC‐derived EVs in promoting PMN rTEM. Herein, analyzing single‐cell RNA sequencing (scRNA‐seq), the rTEM PMN subgroup was initially identified in the lung of mice subjected to cecal ligation and puncture (CLP), a model of polymicrobial sepsis. Next, the association between rTEM PMNs and remote lung injury was investigated through clinical and in vivo assays. Finally, the underlying mechanisms of EC‐derived EVs on the PMN rTEM were identified by scRNA‐seq, proteomics techniques, and in vitro and in vivo experiments, which were further confirmed by clinical data related to ARDS.

## 2 Results

### 2.1 scRNA‐seq Analysis of Septic Lung Reveals a Distinct rTEM PMN Subset with Functional Activation

**Figure 1:**
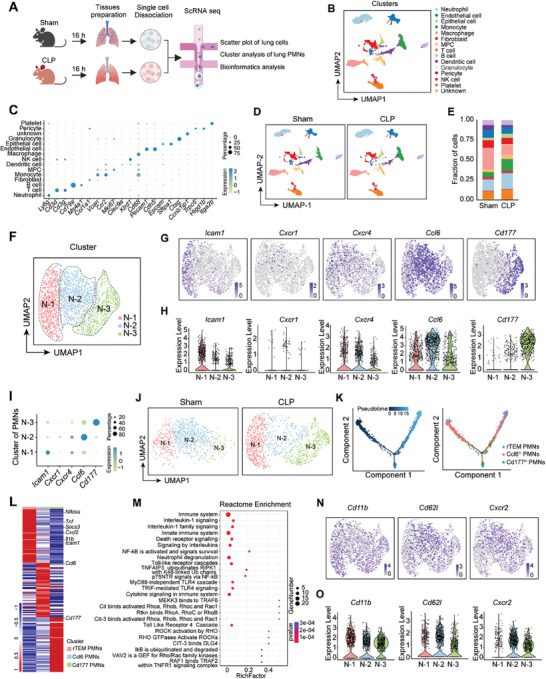
scRNA‐seq analysis of septic lung reveals a distinct rTEM PMN subset with functional activation. A) Schematic of the experimental design (Created with BioRender.com). B) UMAP plot showing the overview of 15 cell clusters of 10979 cells from the mouse lungs of the two groups. C) Dot plot showing the representative marker genes across the 15 clusters. D) UMAP plot showing clusters from the lung of the sham and CLP groups. Clusters are colored as in (B). E) Proportions of the clusters from different groups. Clusters are colored as in (B). F) UMAP plot showing three clusters of pulmonary PMNs. G,H) Gene expression visualized through the UMAP plot (G) and corresponding violin plots (H) for *Icam1, Cxcr1*, *Cxcr4*, *Ccl6*, and *Cd177*. I) Dot plot showing representative marker genes across the three clusters. J) UMAP plot showing the 3 PMN clusters of the lungs from the sham and CLP group. K) Monocle trajectories of PMNs colored by pseudotime (left) and cell subtypes (right). L) Heatmap of enriched genes in each PMN subtype versus the other subsets of pulmonary PMNs. M) Reactome enrichment analysis of the top markers in the rTEM PMN subtype versus the other subsets. N,O) Gene expression visualized by UMAP plot (N) and corresponding violin plots (O) for *Cd11b*, *Cd62l*, and *Cxcr2*.

scRNA‐seq has emerged as a powerful tool for subpopulation analysis and for delineating the molecular profiles of individual cells. To analyze rTEM PMNs in the lungs during sepsis, we performed scRNA‐seq on lung tissues from mice subjected to sham or CLP using the 10X platform (**Figure** **1**A). After rigorous quality control 10979 cells were analyzed for transcriptional profiling, including 5329 cells from the sham‐treated mice and 5650 cells from the CLP‐treated mice (Figure S1A–C, Supporting Information). By using nonlinear dimensional reduction and uniform manifold approximation and projection (UMAP) clustering strategies, the lung cells were clustered into 15 discrete cell populations, namely neutrophil (*Ly6g*), EC (*Pecam1, Cdh5*), epithelial cell (*Epcam, Sftpa1*), monocyte (*Ccr2*, *Vcan*), macrophage (*Cd68*), fibroblast (*Col1a1*), mesenchymal progenitor cell (MPC; *Mki67*), T cell (*Cd3d, Cd3g*), B cell (*Cd79a, Ms4a1*), dendritic cell (*Clec9a*), granulocyte (*Ctsg*), pericyte (*Higd1b, Trpc6*), NK cell (*Klrd1*), platelet (*Itga2b*), and unknown (*Ccnb1ip1*) (Figure 1B,C). Notably, we observed an elevated proportion of PMNs in the lungs of CLP‐operated mice compared to that in sham mice (Figure 1D,E).

The rTEM PMN phenotype has well‐established surface markers with high ICAM1 expression and low CXCR1 expression (ICAM1^hi^CXCR1^lo^) and high CXCR4 expression (CXCR4^hi^).^[^ ^9^, ^17^ ^]^ Next, we aimed to identify the rTEM PMN subset in septic lungs according to the expression distribution of these markers above. We classified the1866 lung PMNs from the two groups into three major PMN subpopulations: N‐1 (highly expressed *Icam1*, *Cxcr4* and lowly expressed *Cxcr1*), defined as rTEM PMNs; N‐2 (highly expressed *Ccl6*), defined as Ccl6^hi^ PMNs; and N‐3 (highly expressed *Cd177*), defined as Cd177^hi^ PMNs (Figure 1F–I). An increased proportion of rTEM PMNs was observed in lungs after CLP compared to that in sham mice (Figure 1J). Pseudo‐time trajectory analysis indicated that the Ccl6^hi^ subset was the root of the trajectory, and rTEM PMNs were in end‐point states (Figure 1K). Substantial differential gene expression was observed among these subsets (Figure 1L). To characterize the functional activation of rTEM PMNs in septic lungs, we performed Reactome Enrichment analysis of the top markers in the rTEM PMN subset compared to the other two subsets. The most enriched functional pathways were inflammatory signal transduction pathways, including interleukin‐1 signaling, toll‐like receptor cascades, and NF‐κB activation and signal survival, suggesting their functional activation and enhanced survival capacity of rTEM PMNs (Figure 1M). Moreover, rTEM PMNs in septic lungs had higher surface expression of the activation indicator *Cd11b*,^[^ ^18^ ^]^ and lower expression of *Cd62l* and *Cxcr2* (Figure 1N,O), which are negative indicators of PMNs activation.^[^ ^18^, ^19^ ^]^ Collectively, these findings indicate a prominent increase in rTEM PMN subsets with predominantly activated phenotype in the lungs of septic mice.

### 2.2 Heightened Proportion of rTEM PMNs Positively Correlates with the Disease Severity in Patients with Sepsis and in Septic Mice

**Figure 2:**
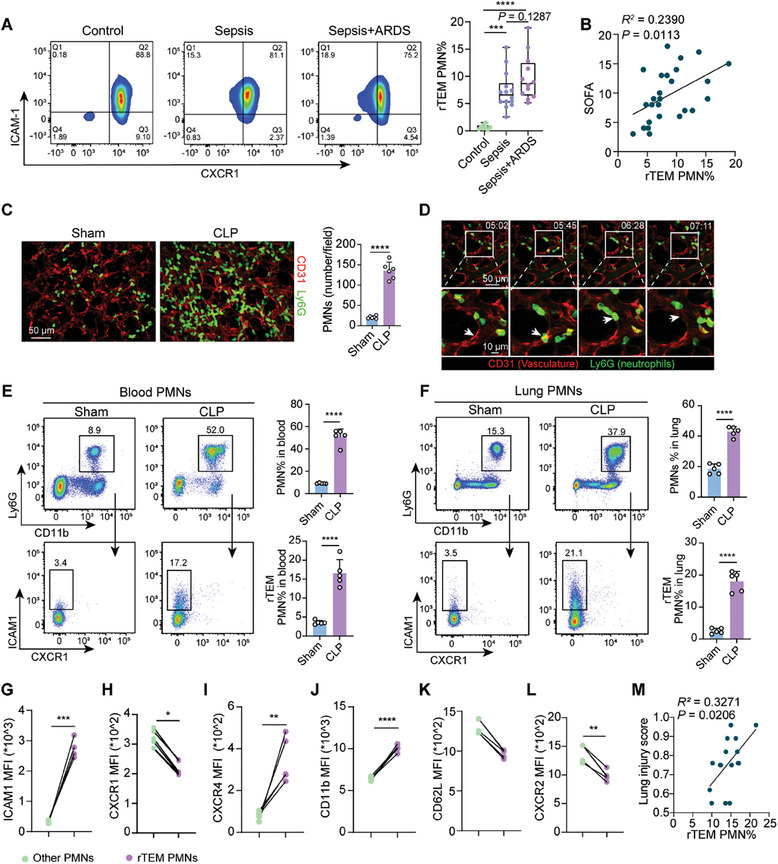
Heightened proportion of rTEM PMNs positively correlates with the disease severity in patients with sepsis and in septic mice. A) The proportion of rTEM PMNs in peripheral PMNs (CD66b+) from healthy control (n = 8), sepsis patients with or without ARDS (n = 12–14) within 48 h after diagnosis. B) Correlation of the proportion of rTEM PMNs in blood with SOFA score in patients with sepsis (n = 24). C) Representative intravital imaging of PMNs labeled by Ly6G (green) and pulmonary microcirculation labeled by CD31 (red) in mice 12 h after operation (Movies S1 and S2, Supporting Information). Quantification of PMNs in the lung, twe fields of view per mouse (n = 3). Scale bar, 50 µm. D) Time‐lapse confocal images showing a PMN rTEM event in the pulmonary vasculature of CLP‐operated mice with the PMN (white arrow) in the subendothelial space (t = 05: 45 min) re‐entering the vascular lumen (t = 06: 28 min) (Movie S3, Supporting Information). Scale bars, 50 µm (wide field) and 10 µm (magnified spot). E,F) Flow cytometric analysis of PMNs and rTEM PMNs in the blood (E) and lungs (F) of mice euthanized 12 h (n = 5). G–L) Mean fluorescence intensity (MFI) of ICAM1, CXCR1, CXCR4, CD11b, CD62L, and CXCR2 in rTEM PMNs compared to the others in pulmonary PMNs of CLP‐operated mice (n = 5). M) Correlation analysis between pulmonary rTEM PMN proportions and the lung injury score at 12 h (n = 16). Statistics: one‐way ANOVA with Dunnett's multiple comparison test in (A); unpaired two‐tailed *t‐test* or two‐tailed Mann–Whitney *U‐test* in (E, F); Pearson's correlation coefficient analysis in (B, M); Wilcoxon match‐paired two‐tailed signed rank test in (G–L). Data are represented as mean ± SEM. ns, no significance. *P < 0.05, **P < 0.01, ***P < 0.001, and ****P < 0.0001.

Given the association between sepsis and increased vulnerability to lung dysfunction, we evaluated the proportion of rTEM PMNs in the clinic. We first investigated the frequency of rTEM PMNs in the bloodstream of patients with sepsis compared to that in healthy controls. As previously documented, a minor subset of PMNs characterized by the rTEM phenotype is consistently present in healthy adults.^[^ ^7^ ^]^ Of significance, the proportion of rTEM PMNs in patients with sepsis was elevated compared to control volunteers (**Figures** **2**A and S2A, Supporting Information), as identified by their distinct phenotype markers. Moreover, patients with sepsis who developed ARDS exhibited a higher proportion of rTEM PMNs in the periphery than those who were not diagnosed with ARDS (Figure 2A). Importantly, the proportion of PMNs undergoing rTEM positively correlated with the subsequent severity of multiple organ failure, as evaluated by the sequential organ failure assessment (SOFA) score within 48 h of ICU admission (Figure 2B). These findings suggest a potential association between rTEM PMNs and the development of lung damage in patients with sepsis.

To directly test whether reverse transendothelial trafficking of PMNs occurs in mice exposed to CLP, confocal intravital microscopy (IVM) was used to analyze the dynamics of PMNs located in the pulmonary microcirculation as well as in the intestinal vascular system, considering the initial infectious site of the intestine. The vessels and PMNs were labeled by intravenous injection of FITC‐conjugated CD31 and PE‐conjugated Ly6G antibodies, respectively. The inflammatory injury model exhibited profound PMN infiltration (Figure 2C). Real‐time intravital imaging of the pulmonary capillaries showed transient entrapment of PMNs in the capillaries during circulation in sham mice, whereas PMN recruitment was notably augmented in the mouse model of sepsis (Figure 2C; Movies S1 and S2, Supporting Information). PMNs engaging in rTEM first approached the blood vessel, migrated to the subendothelial space, reentered, and eventually were removed by the bloodstream (Figure 2D; Movie S3, Supporting Information). While rTEM has been observed in various parts of the body, confocal intravital imaging results from the present study are the first to suggest that this process occurs in the pulmonary blood vessels (Figure 2D) as well as in the intestinal vascular system during sepsis (Figure S3A, Supporting Information).

Next, we performed a quantitative assessment of PMNs and rTEM PMNs in the systemic circulation and lung tissues of septic mice using flow cytometry. The proportions of PMNs and rTEM PMNs increased at 12 h in the blood and lungs of mice subjected to CLP compared to the sham mice (Figures 2E,F and S4A,B, Supporting Information). Notably, the phenotype of rTEM PMNs was analyzed using flow cytometry (Figure 2G–I). PMNs in septic lungs exhibited higher expression of CD11b and lower expression of CD62L and CXCR2, indicating a predominantly activated phenotype (Figure S4C–E, Supporting Information). In addition, rTEM PMNs showed a more functionally activated phenotype than the other PMNs in the pulmonary PMNs of septic mice (Figure 2J–L). Furthermore, the proportion of pulmonary rTEM PMNs was positively correlated with lung injury scores (Figure 2M).

Given the impact of sex differences on the immune response,^[^ ^20^ ^]^ we further explored the effect of sex differences on lung rTEM PMNs and survival in septic mice. We analyzed PMN and rTEM PMN subpopulations in the lungs of male and female mice after CLP using flow cytometry. Twelve hours after CLP‐induced sepsis, both male and female mice showed an increased proportions of pulmonary PMN and rTEM PMN compared to control mice. Although the proportions of PMN and rTEM PMN in the lungs of male mice were higher than those in female mice subjected to CLP, these differences were not statistically significant (Figure S5A, Supporting Information). Additionally, PMN activation was similar between male and female septic animals, as evidenced by the expression of ICAM1, CD11b, CD62L, and CXCR2 measured by flow cytometry (Figure S5B, Supporting Information). Moreover, similar survival rates were observed between male and female mice following CLP‐induced sepsis (Figure S5C, Supporting Information), which was consistent with previous animal studies.^[^ ^21^ ^]^ These results indicate that sex differences have a minimal impact on rTEM PMNs and survival in septic mice undergoing CLP surgery.

Collectively, these findings demonstrate that the proportion of rTEM PMNs is significantly increased in sepsis, which probably contributes to subsequent lung injury.

### 2.3 Adoptive Transfer of rTEM PMNs Aggravates Pulmonary Injury

**Figure 3:**
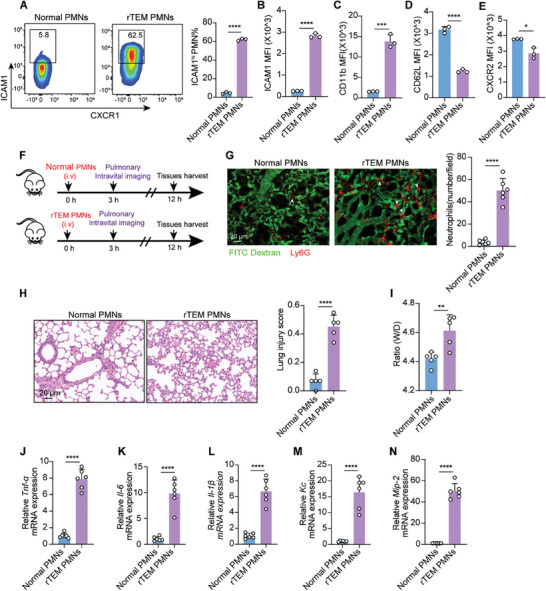
Adoptive transfer of rTEM PMNs aggravates pulmonary injury. A,B) Generation of rTEM PMNs in vitro (A) and ICAM1 expression on normal PMNs or rTEM PMNs (B) (n = 3). C–E) MFI of CD11b, CD62L, and CXCR2 expression on normal PMNs or rTEM PMNs (n = 3). F) Schematic showing the administration of rTEM PMNs, intravital imaging and tissue harvest. G) Representative intravital imaging of Ly6G‐labeled normal PMNs (red) or rTEM PMNs (red) and pulmonary microcirculation (FITC dextran, green) (Movies S4 and S5, Supporting Information). Quantification of PMN infiltration in lungs 3 h after PMN transfusion (two fields of view per mouse, n = 3). Scale bar, 20 µm. H) Representative images of H&E‐stained lung sections in mice and lung injury score 12 h after treatment (n = 5). Scale bar, 20 µm. I) W/D ratio of lung tissues of mice with indicated intervention after 12 h (n = 5). J–N) qPCR analysis of *Tnf‐α, Il‐6, Il‐1β, Kc*, and *Mip‐2* in the lung of mice 12 h after PMN transfusion (n = 6). Statistics: unpaired two‐tailed *t‐test* in (A–E; G–N). Data are represented as mean ± SEM. *P < 0.05, **P < 0.01, ***P < 0.001, and ****P < 0.0001.

To address the contribution of rTEM PMNs to pulmonary inflammation and injury, we conducted adoptive cell transfer experiments to investigate the potential effect of rTEM PMNs on lung damage. According to a previously established method,^[^ ^9b^ ^]^ we generated a population of more than 60% PMNs with rTEM phenotype of high ICMA1 expression (ICAM1^hi^) under TNF‐*α* stimuli (**Figure** **3**A,B). The rTEM PMNs generated in vitro exhibited an activated phenotype with higher CD11b and lower CD62L and CXCR2 expression than normal PMNs (Figure 3C–E). Moreover, in agreement with previous studies,^[^ ^7^, ^9^ ^]^ rTEM PMNs released more reactive oxygen species (ROS) and exhibited greater anti‐apoptotic capacity than normal PMNs (Figure S6, Supporting Information). Next, we employed confocal IVM to monitor pulmonary recruitment of rTEM PMNs after intravenous transfusion. Briefly, rTEM PMNs induced in vitro or normal PMNs were stained with PE‐conjugated anti‐Ly6G anibody and intravenously injected into normal mice (Figure 3F). Employing this methodology, mice injected with normal PMNs demonstrated limited retention of PMNs in the lungs, whereas mice treated with rTEM PMNs showed significant dissemination of PE‐labeled rTEM PMNs in lung tissues after reperfusion (Figure 3G; Movies S4 and S5, Supporting Information). Moreover, in alignment with the increased prevalence of rTEM PMNs, animals treated with these PMNs displayed pronounced lung injury and inflammation, as evidenced by aggravated pulmonary pathology (Figure 3H), elevated wet to dry (W/D) ratio (Figure 3I), and increased gene expression of inflammatory factors and chemokines (Figure 3J–N). Together, these findings indicate that in vivo transfer of reversed transmigrating PMNs is prone to dissemination to the lungs where they become sequestered and subsequently contribute to lung damage.

### 2.4 Analysis of scRNA‐seq Unveils Enhanced Crosstalk Between ECs and PMNs with Elevated EC‐Derived EVs in Sepsis

**Figure 4:**
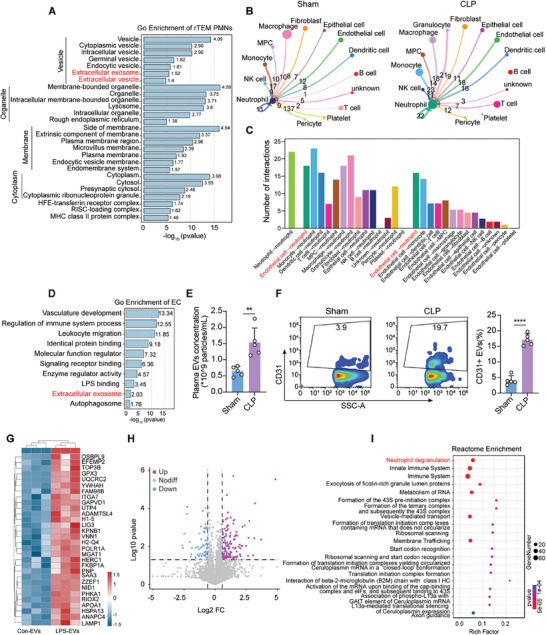
Analysis of scRNA‐seq unveils enhanced crosstalk between ECs and PMNs with elevated EC‐derived EVs in sepsis. A) GO analysis of the top markers in the rTEM PMN subtype versus the other subsets. B) CellChat analysis showing intercellular communication between PMNs and other cells. C) Bar plot depicting the numbers of significant ligand‐receptor interaction between PMNs and the other clusters in the lung of CLP‐operated mice. D) GO analysis of differently expressed genes in ECs from the lung of the CLP group versus the sham group. E) EV particle concentration isolated from equal plasma volumes and determined using Nanoflow cytometer (NanoFCM) (n = 5). F) Quantitative of plasma EVs derived from ECs by identifying CD31 using NanoFCM (n = 5). G) Heatmap of top 30 differentially up‐regulated proteins (avglog2FC > 0.585) in the proteomics data of LPS‐EVs versus Con‐EVs. H) Volcano plot of the differentially expressed proteins (avglog2FC > 0.585) in the proteomics data of LPS‐EVs versus Con‐EVs. I) Reactome enrichment analysis of the proteomics data showing the top 20 pathways enriched in differentially up‐regulated proteins (avglog2FC > 0.585) in LPS‐EVs versus Con‐EVs. Statistics: unpaired two‐tailed *t‐test* in (E, F). Data are represented as mean ± SEM. ***P < 0.001, and ****P < 0.0001.

While rTEM PMNs have been reported to aggravate and propagate inflammation owing to their heightened activation, the mechanism underlying the aberrant modes of neutrophil transendothelial cell migration remains largely unknown. To probe the mechanism underlying PMN rTEM, Gene Ontology (GO) analysis was conducted on the top marker genes of rTEM PMNs identified by scRNA‐seq, suggesting that vehicles may play an important role in this process (**Figure** **4**A). ECs play a crucial role in the PMN extravasation cascade in the early stages of an acute inflammatory response.^[^ ^11a^ ^]^ Therefore, we used CellChat to decipher the crosstalk between ECs and PMNs in the lungs during sepsis. The results showed a significant enhancement in both the number and weight of interactions between ECs and PMNs in the lungs of CLP‐operated mice (Figure 4B,C). To further explore the potential pathways through which ECs influence PMN rTEM, we performed GO analysis of differentially upregulated genes in the ECs of the lungs from the CLP group versus the sham group. Most of the identified biological processes were related to immune regulation, including leukocyte migration and activation. The cellular components involved in extracellular exosome were enriched (Figure 4D).

EC‐derived EVs can be taken up by immune cells to modulate the function of recipient cells.^[^ ^16b^ ^]^ Our previous study demonstrated an augmented release of EC‐derived EVs in ex vivo‐perfused human lungs with bacterial pneumonia.^[^ ^15^ ^]^ Importantly, EVs have been implicated in the escalation of excessive inflammation. Thus, we conducted in vivo studies to confirm whether EC‐derived EVs increased during sepsis. To this end, plasma EVs were isolated from sham‐operated or CLP‐operated mice by differential centrifugation (Figure S7A, Supporting Information) and subsequently characterized by transmission electron microscopy (TEM), nanoparticle tracking analysis (NTA), and western blotting. The EVs exhibited a round, cup‐shaped morphology with an approximate diameter of 100 nm, as confirmed by TEM and NTA (Figure S7B,C, Supporting Information). Western blotting revealed elevated expression of EV‐specific markers, such as CD63, CD9, TSG101, and Alix, whereas negative markers, including GM130 and Calnexin, were absent in both sham and septic mice samples (Figure S7D, Supporting Information). Furthermore, septic mice exhibited a heightened total particle concentration of circulating EVs compared to sham mice when isolated from equivalent plasma volumes (300 µL) (Figure 4E), as well as a significant increase in EC‐derived EVs, as detected by an EC marker CD31, contrary to the sham sample (Figure 4F). To better analyze the potential role of EC‐derived EVs in PMN rTEM, mass spectrometry was conducted on EVs isolated from the supernatant of ECs stimulated with either vehicle (Con‐EVs) or LPS (LPS‐EVs) for 24 h (Figure 4G). Isolation was performed through differential ultracentrifugation and characterized by TEM, NTA, and western blotting (Figure S7E–I, Supporting Information). The heatmap and volcano plots of the mass spectrometry of EVs were analyzed (Figure 4G,H). Functional enrichment analysis of the differentially upregulated proteins by mass spectrometry predominantly revealed a significant enrichment in the neutrophil degranulation pathway (Figure 4I), which has been reported to be involved in promoting the transendothelial migration of PMNs as well as cancer cells.^[^ ^22^ ^]^ Therefore, we speculate that EVs are a plausible mechanism for the involvement of EC‐derived EVs in the rTEM process of PMNs during sepsis.

### 2.5 EC‐Derived EVs Program PMNs Toward rTEM PMN Phenotypes In Vivo and Cause Remote Lung Injury

**Figure 5:**
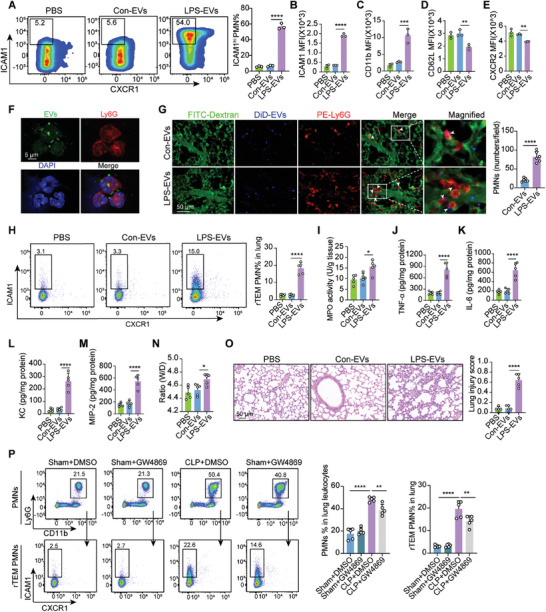
EC‐derived EVs program PMNs toward rTEM PMN phenotypes in vivo and cause remote lung injury. A) Comparisons of the proportion of rTEM PMNs induced by phosphate buffered saline (PBS), Con‐EVs, and LPS‐EVs using flow cytometry (n = 3). B–E) MFI of ICAM1, CD11b, CD62L, and CXCR2 expression in PMNs treated with indicate treatment (n = 3). F) Colocalization of GFP‐labeled EVs (green) and Ly6G (red) after incubation for 3 h. G) Representative intravital imaging of pulmonary microcirculation (FITC dextran, green), DiD‐labeled EVs (blue), and PE‐labeled Ly6G (red) in mice treated with Con‐EVs and LPS‐EVs (Movies S6 and S7, Supporting Information). Scale bar, 50 µm. Quantification of PMN infiltration in lungs 6 h after treatment (two fields of view per mouse, n = 3). H) Flow cytometry analysis of rTEM PMN proportions in the lungs of mice at 12 h (n = 5). I) MPO activity of lung tissues at 12 h (n = 5). J–M) Protein expression of TNF‐*α*, IL‐6, KC, and MIP‐2 in the lung homogenates (n = 5). N) W/D ratio of lung tissues at 12 h (n = 5). O) Representative images of H&E‐stained lung sections in mice and lung injury score at 12 h (n = 5). P) Flow cytometry analysis of rTEM PMN proportions on the lungs in septic mice pretreated with GW4869 (n = 5). Statistics: one‐way ANOVA with Dunnett's multiple comparison test (A–E; H–P); unpaired two‐tailed *t‐test* in (G). Data are represented as mean ± SEM. *P < 0.05, **P < 0.01, ***P < 0.001, and ****P < 0.0001.

To determine whether EC‐derived EVs promote the rTEM PMN phenotype, we previously established an immortalized cell line of mouse pulmonary EC as a stable EC‐derived EVs source.^[^ ^23^ ^]^ Compared to Con‐EVs, mouse bone marrow‐derived PMNs stimulated by LPS‐EVs exhibited a reversal of transmigration through cultured ECs, which exhibited a higher percentage of ICAM1^hi^ PMNs (**Figure** **5**A), and showed higher ICAM1, CD11b expression, and lower CD62L, CXCR2 expression (Figure 5B–E). EVs with green fluorescent protein (GFP) signals were obtained from stably CD63‐GFP overexpressed ECs. The co‐localization of GFP‐labeled EVs and Ly6G suggested the EVs uptake capacity of PMNs (Figure 5F).

Next, to gain further insights into the role of EC‐derived EVs in PMN rTEM and lung injury in vivo, we intravenously transfused LPS‐EVs, which mimic EV production during sepsis in vivo, into normal mice according to previous methods.^[^ ^24^ ^]^ First, we observed the in vivo biodistribution of LPS‐EVs labeled with fluorescent probe DiD after intravenous EVs administration to healthy mice. DiD fluorescence was predominantly observed in the livers and lungs at 6 h, whereas pulmonary DiD fluorescence gradually increased and peaked 24 h after injection (Figure S8A, Supporting Information). To better visualize EVs uptake by PMNs in the lungs in *vivo*, confocal IVM of pulmonary vascular imaging was performed. Mice were intravenously injected with DiD‐labeled EVs, followed by 200 kD FITC‐Dextran and PE‐conjugated anti‐Ly6G antibody to visualize blood vessels and PMNs. We noted that within a few hours of intravenous EV injection, most EVs were internalized by PMNs, coinciding with a notable increase in the number of PMNs in the lungs after LPS‐EVs treatment (Figure 5G; Movies S6 and S7, Supporting Information). Subsequently, flow cytometry analysis demonstrated that LPS‐EVs significantly increased the proportion of rTEM PMNs and induced lung MPO activity compared with the Con‐EVs (Figure 5H,I). Lung tissue immunofluorescence assays revealed that LPS‐EVs significantly increased the number of Ly6G‐positive cells and ICAM1‐positive PMNs compared with Con‐EVs (Figure S8B, Supporting Information). Importantly, the treatment with LPS‐EVs resulted in increased levels of inflammatory factors and chemokines (Figure 5J–M), an elevated W/D ratio (Figure 5N), and significant pathological lung damage (Figure 5O) compared to those receiving Con‐EVs. In addition, inhibition of EV production with GW4869, a commonly used chemical inhibitor of EV/exosome biogenesis, prior to the CLP model, markedly reduced PMN infiltration and rTEM PMN proportions in the lungs of CLP‐subjected mice compared to sham mice, as demonstrated by flow cytometry and immunofluorescence staining (Figures 5P and S9A,B, Supporting Information). Consistent with these findings, GW4869 administration significantly lowered MPO activity (Figure S9C, Supporting Information), pulmonary inflammation, and pathological changes (Figure S9D–I, Supporting Information). Moreover, GW4869 treatment increased the survival rate of septic mice (Figure S9J, Supporting Information). In addition, lung function assessment demonstrated that GW4869 ameliorated lung dysfunction in septic mice, as indicated by the respiratory frequency, tidal volume, peak expiration height, and Penh (Figure S9K, Supporting Information). Taken together, EC‐derived EVs in an inflammatory context increased the proportion of rTEM PMNs in the lungs, ultimately contributing to lung injury.

### 2.6 LPS‐EVs Support PMN rTEM through the Upregulation of NE and Subsequent Degradation of JAM‐C

**Figure 6:**
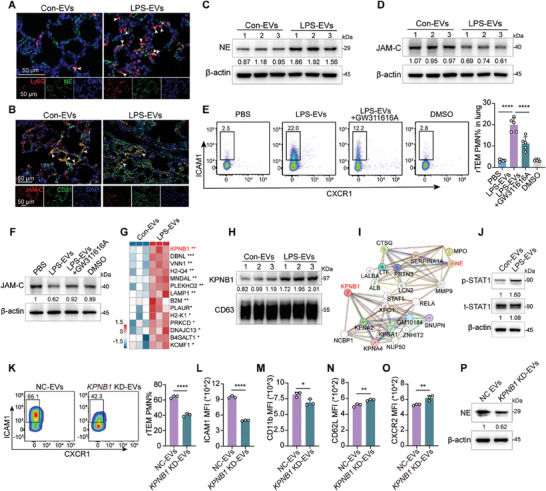
LPS‐EVs support PMN rTEM through the upregulation of NE and subsequent degradation of JAM‐C. A) Representative immunostaining images for NE (green) and Ly6G (red). White arrows point to the co‐location of the two markers. Scale bar, 50 µm. B) Representative immunostaining images for CD31 (green) and JAM‐C (red). White arrows point to the co‐location of the two markers. Scale bar, 50 µm. C,D) Western blotting analysis of NE and JAM‐C in the lungs of mice injected with Con‐EVs or LPS‐EVs. E) Flow cytometric analysis of pulmonary rTEM PMN proportion in LPS‐EVs injected mice pretreated with GW311616A (n = 5). F) Western blotting analysis of JAM‐C expression in the lung of LPS‐EVs treated mice with NE inhibitor GW311616A pretreatment. G) Heatmap of 14 proteins within the enriched neutrophil degranulation pathway analyzed in (Figure 4I) of the proteomics data. H) Western blotting analysis of KPNB1 expression in Con‐EVs versus LPS‐EVs (n = 3). I) PPI analysis between KPNB1 and NE. J) Western blotting analysis of p‐STAT1 in the lung of LPS‐EVs treated mice. K) Flow cytometric analysis of rTEM PMNs after treated with EVs from the cell supernatant of *KPNB1*‐knockdown ECs (n = 3). L–O) MFI of ICAM1, CD11b, CD62L, and CXCR2 in rTEM PMNs after treatment with NC‐EVs or *KPNB1* KD‐EVs (n = 3). P) Western blotting analysis of NE in PMNs treated with *KPNB1* KD‐EVs or NC‐EV. Statistics: one‐way ANOVA with Dunnett's multiple comparison test in (E); unpaired two‐tailed *t‐test* in (K–O). Data are represented as mean ± SEM. *P < 0.05, **P < 0.01, ***P < 0.001, and ****P < 0.0001.

Next, we aimed to elucidate the mechanism by which LPS‐EVs trigger PMN rTEM. Previous investigations have demonstrated that neutrophil elastase (NE), one of the major granules involved in neutrophil degranulation, promoting PMN rTEM through junctional adhesion molecule‐C (JAM‐C) degradation.^[^ ^9^, ^10^, ^25^ ^]^ Moreover, analysis of EV mass spectrometry showed significant enrichment in neutrophil degranulation pathway (Figure 4I). In line with earlier findings,^[^ ^26^ ^]^ we observed increased NE protein expression and decreased JAM‐C expression in the lungs of CLP‐operated mice relative to those of sham mice, as demonstrated by western blotting (Figure S10A,B, Supporting Information). Therefore, we postulated that LPS‐EVs induce PMN rTEM by upregulating NE protein, which leads to JAM‐C degradation at EC junctions. Similarly, administration of LPS‐EVs resulted in the upregulation of NE protein expression in PMNs and a significant reduction in JAM‐C at EC junctions compared to Con‐EVs, as detected by immunofluorescence and western blotting (**Figure** **6**A–D). Furthermore, pretreatment with the NE inhibitor GW311618A reduced the proportion of pulmonary rTEM PMNs (Figure 6E) and attenuated JAM‐C degradation by LPS‐EVs (Figure 6F). These findings suggest the involvement of NE‐mediated JAM‐C cleavage in the promotion of rTEM processes upon LPS‐EVs treatment.

Next, we investigated the specific mechanism through which LPS‐EVs initiate alterations in NE expression and the PMN phenotype. After enrichment analysis using EV mass spectrometry, 14 differentially upregulated proteins associated with neutrophil degranulation pathway were visualized using a heatmap representation (Figure 6G). Among these candidate proteins, karyopherin subunit beta‐1 (KPNB1), a pivotal nuclear receptor involved in facilitating the translocation of proteins from the cytoplasm to the nucleus, including p65, activates the NF‐κB pathway and are linked in the pathogenesis of inflammation amplification.^[^ ^27^ ^]^ Consequently, we validated whether LPS‐EVs contribute to this process by delivering KPNB1 cargo, potentially enhancing the expression of serine proteases within PMN azurophil granules. LPS‐EVs significantly increased KPNB1 protein levels (Figure 6H). KPNB1 has been implicated in increased signal transducer and activator of transcription 1 (STAT1) expression.^[^ ^28^ ^]^ Moreover, protein–protein interaction (PPI) analysis between KPNB1 and NE indicated a potential interaction facilitated by STAT1 activation (Figure 6I), and STAT1 phosphorylation in lung was significantly activated by LPS‐EVs treatment (Figure 6J). Based on the above, we postulate that KPNB1‐containing LPS‐EVs facilitate NE upregulation in PMNs, probably by STAT1 activation, subsequently causing PMN rTEM.

Next, we established *KPNB1*‐knockdown cell line in mouse ECs to determine the role of KPNB1 in LPS‐EVs (Figure S11A, Supporting Information). Subsequently, EVs were obtained from *KPNB1*‐knockdown cells *(KPNB1* KD‐EVs) or control vector‐treated cells (NC‐EVs), stimulated with LPS for 24 h and the protein expression of KPNB1 and CD63 were characterized by western blotting (Figure S11B, Supporting Information). Flow cytometry analysis revealed that PMNs pretreated with *KPNB1* KD‐EVs exhibited a notably decreased proportion of rTEM PMNs (Figure 6K), accompanied by a significant reduction in ICAM1 and CD11b expression, and increased CD62L and CXCR2 expression compared to NC‐EVs (Figure 6L–O). Additionally, a lower NE expression in PMNs was observed after *KPNB1* KD‐EVs treatment in comparison to NC‐EVs (Figure 6P). Collectively, these results indicate that the KPNB1 cargo of LPS‐EVs may promote PMN rTEM by upregulating NE via STAT1 activation.

### 2.7 Plasma EC‐Derived EVs are Increased in Patients with Sepsis‐Associated ARDS

**Figure 7:**
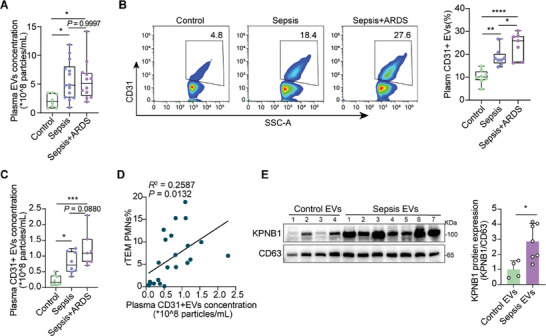
Plasma EC‐derived EVs are increased in patients with sepsis‐associated ARDS. A) Particle concentration of EVs from equal plasma volumes of healthy control (n = 8) and sepsis patients with or without ARDS (n = 26). B) Proportion of EC‐derived EVs identified in the plasma of sepsis patients with or without ARDS (n = 8–15). C) Particle concentrations of CD31+ EVs in the plasma of sepsis patients with or without ARDS. D) Correlation of the plasma CD31+ EVs particle concentration with rTEM PMN proportions in peripheral in individuals from (C) (n = 23). E) Western blotting analysis of KPNB1 and CD63 in EVs from the plasma of patients with sepsis. Right, quantification of grey value. (healthy control volunteers, n = 4; sepsis patients with or without ARDS, n = 7). Statistics: one‐way ANOVA with Dunnett's multiple comparison test in (A–C); Pearson's correlation coefficient analysis in (D); unpaired two‐tailed *t‐test* in (E). Data are represented as mean ± SEM. ns, no significance. *P < 0.05, ***P < 0.001, and ****P < 0.0001.

To further explore the clinical implications of our findings, we assessed EV concentrations in the plasma of patients with sepsis. In our study, the plasma EV concentrations of total EVs were significantly elevated in patients with sepsis with or without ARDS compared to those in healthy controls (**Figure** **7**A). Importantly, the proportion of EVs derived from ECs, identified by CD31, was higher in patients with sepsis than that in control volunteers by NanoFCM (Figure 7B). The particle concentrations of CD31‐positive EVs (CD31+ EVs) in the plasma of sepsis patients with ARDS were higher than those in sepsis patients without ARDS; however, no significant differences were observed between them (Figure 7C). Notably, the plasma CD31+ EVs concentration positively correlated with the rTEM PMN proportion in patients with sepsis (Figure 7D). Moreover, the protein expression of KPNB1 in the plasma EVs of patients with sepsis increased significantly compared to that in controls (Figure 7E). Consequently, elevated levels of EC‐derived EVs and KPNB1 in EVs in the plasma may play a key role in the aberrant migration of PMNs, which may contribute to the development of ARDS in human sepsis.

## 3 Discussion

**Figure 8:**
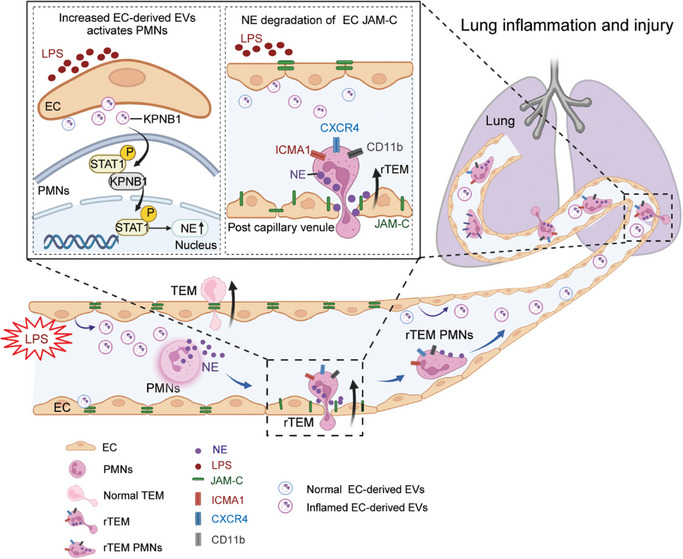
Schematic depicting excessive EC‐derived EVs under inflammatory condition facilitating PMN rTEM and their potential contribution to distant lung inflammation and injury. The diagram illustrates a model depicting the role of rTEM PMNs in the development of septic lung injury. Excessive EVs released from inflamed ECs promoted rTEM of PMNs, resulting in a functionally activated and harmful phenotype. Mechanically, the aberrant response is caused by JAM‐C cleavage at EC junctions by NE, partially mediated by KPNB1 in EC‐derived EVs. Collectively, this proposes that EC‐derived EVs trigger PMN rTEM, which contributes to the development of sepsis‐associated lung injury. The graphics of **Figure** **8** and ToC were created and licensed with BioRender.com.

In this study, we investigated the implications of aberrant modes of neutrophil transendothelial cell migration on distant lung damage in sepsis and the mechanisms of rTEM of PMNs during sepsis. Functionally activated PMNs exhibiting rTEM phenotype were characterized in the lungs of a mouse model of CLP. We demonstrated that the proportion of the rTEM PMN subset was significantly upregulated in patients with sepsis‐associated ARDS and correlated with the severity indicator SOFA score. Moreover, this PMN subset exhibited the capacity for augmented lung retention and lung damage following adoptive transfer. Mechanistically, inflamed ECs remotely activated neutrophil degranulation by releasing EVs, which promoted the rTEM process of PMNs, based on the results of scRNA‐seq and proteomics analysis. Further studies revealed that elevated EC‐derived EVs bearing a cargo rich in KPNB1 stimulate the upregulation of NE protein within PMNs, probably by promoting STAT1 activation. Therefore, PMN rTEM may be the key pathway mediating the onset of remote lung injury in sepsis. To the best of our knowledge, this is the first study to highlight the significance of EC‐derived EVs in driving PMN rTEM process in sepsis‐induced lung injury (Figure 8). The mechanistic insights presented here suggest potential therapeutic avenues for mitigating lung pathologies associated with sepsis and offer enhanced comprehension.

Accumulating evidence has recently indicated significant phenotypic heterogeneity and functional diversity among PMNs, making PMNs essential modulators of both inflammatory and immune responses. Researchers have found that certain PMNs that initiate TEM exhibit reverse motility within EC junctions and eventually reenter blood circulation, namely, rTEM PMNs.^[^ ^7^, ^12^ ^]^ While the reverse migration of PMNs from inflammatory and injury sites has recently been studied,^[^ ^29^ ^]^ it remains plausible that the outcomes of this process vary in diverse experimental models. In particular, the retrograde migration of PMNs from the interstitial tissues and away from the site of injury in certain instances, is considered a mechanism that contributes to the resolution of inflammation.^[^ ^17^, ^30^ ^]^ In the current study, we provide direct evidence for PMN reverse migration and offer rTEM PMNs as a novel mechanistic component of remote lung damage in sepsis by confocal IVM. Moreover, previous studies have demonstrated that PMNs stemming from sites of hyperpermeability or local inflammatory stimuli reenter the systemic circulation, exhibit an activated phenotype, and remotely traffic to organs.^[^ ^31^ ^]^ Therefore, rTEM processes can be speculated to occur in systematically circulating vessels during sepsis, including the pulmonary vasculature. This was evidenced by the observation of rTEM events in the pulmonary vasculature and intestinal vessels in a mouse sepsis model of CLP. However, we could not calculate the frequency of rTEM events owing to technical limitations. Thus, both the capillarium‐rich pulmonary circulation and the systemic vasculature may be potentially fertile ground for the “rTEM phenotypic shift” of PMNs under a complex inflammatory environment. Based on these results, we conclude that PMNs away from injured or inflamed tissues may be a pathological process involved in the activation and/or reprogramming of PMNs during sepsis. This observation underscores the importance of this phenomenon as a cellular mechanism for aggravating inflammatory reactions in remote organs such as the lungs in sepsis. In addition, immune regulation is often associated with sex differences due to the influence of female sex hormones.^[^ ^20^, ^32^ ^]^ However, our study found no significant difference in rTEM PMNs between male and female septic mice, suggesting that the effect of rTEM PMNs on distant organ injury in the mouse model of sepsis is not significantly related to sex.

However, the mechanisms underlying tissue‐specific migration and retention of PMNs in different organs are poorly understood. In the current study, pulmonary retention and destructive properties of rTEM PMNs were demonstrated using adoptive transfer experiments. This lung retention may be due to the larger diameter of the activated PMN subset than that of the pulmonary capillaries. Dipeptidase‐1 has been reported to be an adhesion regulator of PMNs homing to the lungs; however, whether the expression of this molecule is regulated during sepsis is unknown.^[^ ^33^ ^]^ Additionally, as the alteration in the motion direction of PMNs is instrumental in their functional programming toward an activated and deleterious state, the molecular basis of this process will be of particular interest. The mechanism by which rTEM PMNs induce tissue damage remains to be determined. Future studies should employ mass spectrometry on rTEM PMNs, offering substantial potential to uncover the molecular mechanisms underlying functional alterations, particularly in lung tissues.

Multiple lines of evidence indicate that EVs play an increasingly important role as mediators of intercellular communication in immune regulation.^[^ ^14^, ^34^ ^]^ The highly pro‐inflammatory state during sepsis may additionally induce exaggerated responsiveness of the circulatory endothelium by releasing EVs. ECs generally exert their effects locally within the vasculature and influence the surrounding environment, or directly interact with circulating blood cells. Notably, EC‐derived EVs carry and deliver signaling molecules that mimic EC functions and travel through the bloodstream to distant sites, where remote cells and tissues are influenced by the transfer of cargo to recipient cells. Consistent with investigations in other disease models, including cancer metastatic models and systemic inflammatory diseases, EVs have been shown to contribute to PMN activation and migration.^[^ ^35^ ^]^ In our study, scRNA‐seq analysis revealed upregulation of the EV pathway in rTEM PMNs and enhancement of the interaction between ECs and PMNs. Therefore, we speculate that inflamed ECs may promote PMN rTEM by releasing EVs during sepsis. In vivo and in vitro experiments demonstrated that LPS‐EVs triggered the transformation of PMNs into an rTEM phenotype. Moreover, EV inhibition partly abrogated the induction of PMN rTEM, and to a certain extent, EVs were identified as one of the causal triggers of this aberrant response of PMNs and lung injury. Although we established the promotion of PMN rTEM by EC‐derived EVs, it is plausible that EVs of different cellular origins, such as platelets, could induce analogous effects. Additional investigations are required to validate this hypothesis.

In addressing the molecular mechanisms that trigger PMN rTEM, the sequence of molecular and cellular events that guide PMNs from the vascular abluminal to luminal diapedesis remain to be elucidated, although many studies have reported that rTEM PMNs are significantly elevated in many inflammatory settings.^[^ ^29^ ^]^ Emerging evidence suggests that the NE‐mediated cleavage of EC junctional JAM‐C triggers this cellular response.^[^ ^9b^ ^]^ Combined with in vitro studies of monocyte TEM,^[^ ^25^ ^]^ these findings suggest that EC JAM‐C is a key regulator of unidirectional leukocyte trafficking through EC junctions, although the exact molecular basis of JAM‐C‐mediated luminal‐to‐abluminal PMN movement is unknown. In our study, the loss of JAM‐C and the upregulation of NE in PMNs were also observed in the lungs of mice subjected to CLP or intravenous LPS‐EVs injection, indicating the potential role of EC JAM‐C loss in inducing PMN rTEM in sepsis. Furthermore, the proteomics of LPS‐EVs revealed the notable role of neutrophil degranulation pathway. Previous studies have shown that NE is closely associated with the pathogenesis of several acute and chronic lung diseases.^[^ ^22^, ^36^ ^]^ Based on these results and recent findings, we focused on exploring the potential involvement of EC‐derived EVs in EC JAM‐C degradation by NE, given that NE is predominantly expressed within neutrophil azurophil granules. Supporting this hypothesis, EC‐derived EVs under an inflammatory stimuli demonstrated high efficacy in promoting NE expression, and direct evidence corroborated the role of NE by the administration of an NE inhibitor. Mechanistically, NE upregulation by LPS‐EVs may be attributed to KPNB1, which facilitates the activation of STAT1. Together, our findings shed light on the potent ability of LPS‐EVs to induce the NE upregulation, which emerges as a key factor in the loss of JAM‐C.

The findings of animal experiments yielded similar trends in clinical patients with sepsis‐induced ARDS. Notably, an increased proportion of rTEM PMNs and elevated plasma levels of EC‐derived EVs have been observed in patients with sepsis. The mechanisms underlying the induction of PMN rTEM offer promising targets for therapeutic intervention, potentially involving EC‐derived EVs, KPNB1, and NE. These findings offer novel opportunities to alleviate distant lung damage in patients with sepsis. As the elevated plasma content of EC‐derived EVs has been linked to sepsis‐induced organ failure and is increased in the plasma of patients with trauma and systemic sclerosis, we propose that EC‐derived EVs serve as valuable vascular‐derived biomarkers for assessing the extent of systemic inflammatory responses. Although the exact functional implications of rTEM in PMNs remain unclear, the findings of this study provide additional evidence that links this phenomenon to the dissemination of inflammation.

Our study had several limitations. First, the identification of rTEM PMNs relied on surface phenotype markers, specifically high ICAM1 and CXCR4 expression and low CXCR1 expression, which is consistent with the findings of most previous studies.^[^ ^10^, ^37^ ^]^ Although an advanced in *vivo* cell labeling technique has recently been reported,^[^ ^9^, ^31^ ^]^ providing direct evidence of PMN rTEM occurrence and tracking rTEM PMNs from a local site to the lungs, this method is not applicable to systemic inflammation models such as sepsis. This limitation arises from the technical constraints related to lung implementation and the potential for inaccurate interference. Future research should focus on the development of a more suitable method for tracking rTEM, specifically in various organs. Second, various sources of EVs in the circulation during sepsis other than ECs, such as platelets, epithelial cells, and immune cells.^[^ ^38^ ^]^ Future studies should elucidate whether EVs from other sources are crucial for the initiation of PMN rTEM and induction of lung injury. Third, the lung‐damaging effect of rTEM PMN were demonstrated by rTEM PMNs generated through TNF‐*α* stimulation in vitro, rather than by rTEM PMNs sorted from the peripheral blood or lungs of septic mice using flow cytometry, due to their extreme fragility during the sorting process.^[^ ^19^, ^39^ ^]^ However, further research and advanced technologies are required to verify these finding. Fifth, our investigation of PMNs undergoing rTEM triggered by EC‐derived EVs was primarily evidenced by intravenous administration of LPS‐EVs. We were unable to conclusively demonstrate these results by specifically blocking EV release from ECs in vivo, except for GW4869, owing to the absence of specific pharmacological inhibitors of EC‐derived EVs. Development of inhibitors capable of blocking EV production from ECs will be instrumental in elucidating the contribution of EC‐derived EVs to PMN trafficking and functional activation.

Overall, our study revealed that functionally activated rTEM PMNs potentially establish a detrimental cellular connection linking sepsis to remote lung injury, an axis associated with the development of lung injury in sepsis. We identified the role of EC‐derived EVs containing KPNB1 as regulators of PMN rTEM. This response can facilitate the reentry of PMNs into the vascular lumen, which can contribute to the dissemination of inflammation and the development of lung injury. Collectively, our findings suggest that targeting excessive EC‐derived EVs may be a plausible therapeutic strategy to protect against lung injury in sepsis.

## 4 Experimental Section

### Study Design

The primary objective of this study was to assess the effect of rTEM PMNs on distant lung injury during sepsis and to provide mechanistic insights into the occurrence of PMN rTEM during sepsis. Phenotypic and transcriptomic analysis of rTEM PMNs were performed in patients with sepsis and mice subjected to CLP using flow cytometry, confocal IVM, and scRNA‐seq. Lung damage caused by rTEM PMNs was investigated using adoptive transfer. In vitro and in vivo experiments and EV proteomics were performed to explore the effects of EC‐derived EVs on triggering PMN rTEM. The details of the experimental replicate are provided in the Figure legends.

### Patient Enrollment

All studies and analysis involving human samples conformed fully to the institutional guidelines and received approval from the Ethical Committee of Zhongda Hospital, Southeast University (Approval Number: 2022ZDSYLL402‐Y01). Written informed consent was obtained from all the participants. The patients included in this study met the diagnostic criteria for Sepsis 3.0 because of the etiology of extrapulmonary infection, and were prospectively and randomly recruited from the ICU. Disease activity was evaluated using the SOFA score. Patients were excluded if they 1) were < 18 or > 80 years of age, 2) had been diagnosed with sepsis for > 48 h, 3) had a primary intrapulmonary infection, or 4) had autoimmune or hematologic diseases and had received systemic treatment in past 4 weeks. Controls were recruited from healthy volunteers and matched for sex and age. The demographic information of the participants is presented in Table S1 (Supporting Information).

### Mice

All animal procedures adhered strictly to the National Institutes of Health Guidelines for the Use of Laboratory Animals. The research protocol was approved by Institutional Animal Care and Use Committee at the medical school of Southeast University, Jiangsu, China (Permit Number: 20190222014). Male or female wild‐type C57BL/6J mice, 7–8 weeks old were obtainedfrom GemPharmatech (Nanjing, China). Mice were provided ad libitum access to water throughout the entire experimental period. A 12‐h light and 12‐h dark cycle was maintained for all experiments.

### Mouse Models of Sepsis

The endotoxemia mouse model was established by CLP following established protocols.^[^ ^40^ ^]^ Briefly, the mice were anesthetized, a small midline abdominal incision was mad, and the cecum was exposed. Approximately half of the distal end of the cecum was ligated, and avoid bowel obstruction. An 21‐gauge needle was used to puncture the ligated cecum, with gentle expression of a small amount of feces to ensure puncture patency. The cecum was then returned to the abdominal cavity and the incision was closed. For the sham surgery controls, the cecum was exposed without ligation or puncture. The mice were euthanized at specific time points, and peripheral blood was collected via cardiac puncture for analysis. Lung tissues samples were fixed using a 4% PFA solution and processed for histological staining or immunofluorescence staining. The survival rate was observed 7 days after CLP surgery.

### scRNA‐seq Data Processing and Analysis

Lung samples for scRNA‐seq were collected from sham‐ and CLP‐operated mice. Single‐cell suspensions from the lung tissues were generated using a Lung Dissociation Kit (Miltenyi Biotech, 130‐095‐927, Germany). Cell viability (>95%) was evaluated using trypan blue dye and a hemocytometer. scRNA‐seq was performed on the Chromium platform, using the Single‐Cell 5′ Library and Gel Bead Kit (10×Genomics, 1000169) and Chromium Single‐Cell G Chip Kit (10 × Genomics, 1000120) were used to generate single‐cell gel beads in emulsion (GEMs) following the 10 × Genomics protocol. Libraries were constructed using the Single Cell 5′ Library Kit V2 (10 × Genomics) and sequenced using an Illumina NovaSeq6000 sequencer with a sequencing depth of at least 100 000 reads per cell with a paired‐end 150‐bp reading strategy. The Cell Ranger Software was employed to perform barcode processing and single‐cell 5′unique molecular identifier (UMI) counting. Cell barcodes were subsequently determined based on the distribution of UMI counts. Each cell in the sample met the following criteria: gene number >200 and mitochondrial gene percentage <0.2. After filtration, 10979 cells remained for subsequent analysis. Finally, a filtered gene‐barcode matrix for all samples was integrated using Seurat 4.0.2. The first 50 dimensions of principal‐component analysis (PCA) were utilized in the parameter settings. Harmony was employed to correct batch effects in the PCA space during clustering of major cell lineages.

### Confocal Intravital Microscopy (IVM)

A custom‐built laser‐scanning confocal microscope modified from a previously constructed system (IVIM Technology) was used to visualize PMN recruitment and trafficking following previously methods.^[^ ^41^ ^]^ Briefly, mice were anesthetized using 4% isoflurane at a flow rate of 500 mL min^−1^. Anesthetized mice received an intravenous injection of 20 µg FITC‐conjugated anti‐mouse CD31 anibody or 2000 kDa FITC‐conjugated Dextran (40 mg kg^−1^, FD2000S, Sigma–Aldrich) to label vessels within the tissues, and 25 µg of PE‐conjugated anti mouse Ly6G anibody to specifically label the PMNs 30 min before imaging. To visualize the mode and dynamics of PMN migration in the lung or intestine, time‐lapse images were taken at 30‐s intervals after injection. For in vivo imaging of EVs uptake by PMNs, purified EVs fluorescently labeled with DiD were intravenously injected into mice with a 31‐G microinjector Time‐lapse images were taken at 30‐s intervals after injection. The accumulated PMNs in observed tissues per field of view were quantified by manual counting at the end of the IVM imaging period. The proportion of EVs uptake by PMNs was quantified by manual calculation of positive co‐labeling of DiD with Ly6G.

### Nanoflow Cytometer

NanoFCM (Xiamen, China) was used to analyze the EV preparations and determine the particle concentration, size distribution, and surface protein makers according to established protocols. To determine the proportion of EC‐derived EVs in the plasma of humans and mice, EV samples were mixed with FITC‐conjugated Anti‐human CD31 or FITC‐conjugated anti‐mouse CD31 anibodies, respectively, according to the manufacturer's instructions. This mixture was then incubated at 37 °C for 60 min, washed with PBS, and subsequently centrifuged at 100 000 × g for 1.5 h. The resulting pellets were analyzed using NanoFCM and FlowJo V10 software (Tree Star, Inc., Ashland, OR, USA).

### Mass Spectrometry

Proteomic analysis of EVs was conducted by EVbio Technology Co., Ltd. (Beijing, China). The collected EVs were lysed using lysis buffer containing protease inhibitors and subsequently centrifuged at 12000 × g for 30 min to collect the protein supernatant. For the analysis, an equivalent amount of 100 µg protein from both Con‐EVs and LPS‐EVs was analyzed using an ORBITRAP ECLIPSE mass spectrometer (Thermo Fisher Scientific) equipped with a nanoelectrospray ion source (Nanospray Flex). The raw files were processed using Proteome Discoverer 2.4 (Thermo Fisher Scientific) against the Mus musculus proteome database. The parameters were conFigured as follows: the enzyme specificity was set to trypsin; the protein modifications included carbamidomethylation (C), M oxidation, and acetyl (protein N‐terminal); a maximum of two missed cleavages was allowed; the precursor ion mass tolerance was defined as 15 ppm; and the fragment ion mass tolerance was 0.02 Da. Heatmap, Reactome enrichment, and GO analysis were performed using the OmicShare tool (https://www.omicshare.com/tools). PPI analysis was conducted using the STRING database (https://string‐db.org/).

### Statistical Analysis

Statistical analysis were performed using GraphPad Prism 9.0 (GraphPad Software, San Diego, CA, USA). Two‐tailed paired and unpaired Student's *t‐tes*t or two‐tailed Mann–Whitney *U‐test* were employed to assess the statistical significance between the two groups. One‐way ANOVA with Dunnett's multiple comparison test or two‐way ANOVA with Sidak's multiple comparison test were performed for multiple comparisons as appropriate. Pearson's correlation coefficient was used to quantify correlations. Survival was compared using log‐rank analysis. The *chi*‐squared test was used to compare categorical variables. All data were presented as the mean of at least three independent replicates. The data were presented as the mean ± SEM (standard error of the mean). Data were considered statistically significant when *P* value < 0.05. *P < 0.05, **P < 0.01, ***P < 0.001, and ****P < 0.0001 compared to control.

## Conflict of Interest

The authors declare no conflict of interest.

## Author Contributions

S.‐F.Z., X.‐J.W., and Y.T. contributed equally to this work. S.‐F.Z., X.‐J.W., Y.T., J.C., and H.‐B.Q. designed the experiments. S.‐F.Z., Y.‐P.L., X.L., L.W., S.‐L.L., C.‐D.W., and J.‐Y.X. conducted the experiments. T.L., W.H., J.‐F.X., J.C., L.L., and H.‐B.Q. examined and interpreted the data. S.‐F.Z., X.‐J.W., and Y.T. drafted the paper, and S.‐F.Z., J.C., and H.‐B.Q. revised the manuscript. All authors read and approved the final manuscript.

## Supporting information

Supporting Information

Supplemental Movie 1

Supplemental Movie 2

Supplemental Movie 3

Supplemental Movie 4

Supplemental Movie 5

Supplemental Movie 6

Supplemental Movie 7

## Data Availability

The data that support the findings of this study are available from the corresponding author upon reasonable request.
