# Supplementary material for: Endothelial Cell‐Derived Extracellular Vesicles Promote Aberrant Neutrophil Trafficking and Subsequent Remote Lung Injury
Source: Adv Sci (Weinh). 2024 Aug 9;11(38):2400647. doi: 10.1002/advs.202400647 (PMC11481253; doi:10.1002/advs.202400647)
Supplement: Supplementary file 1 — Supporting Information [file ADVS-11-2400647-s003.docx]

Supporting Information

**Endothelial Cell-Derived Extracellular Vesicles Promote Aberrant Neutrophil Trafficking and Subsequent Remote Lung Injury**

*Shuang-Feng Zi^1†^, Xiao-Jing Wu^1†^, Ying Tang^1†^, Yun-Peng Liang^1^, Xu Liu^1^, Lu Wang^1^, Song-Li Li^1^, Chang-De Wu^1^, Jing-Yuan Xu^1^, Tao Liu^1,3^, Wei Huang^1^, Jian-Feng Xie^1^, Ling Liu^1^, Jie Chao^1,2*^ Hai-Bo Qiu^1*^*

^†^ These authors contributed equally

^*^ Corresponding author

**This file includes:**

Supplementary Methods

Figures S1 to S11

Tables S1 to S6

Movies S1 to S7

**Supplementary Methods**

*Lung histology and quantification*: The left lung lobes were fixed in 4% paraformaldehyde and subsequently embedded in paraffin. Fixed tissues were sliced into 5 μm thick sections and stained with hematoxylin and eosin (H&E). To evaluate the lung injury score, 6 randomly chosen fields were evaluated according to five independent variables: PMNs in the alveolar space, PMNs in the interstitial space, hyaline membranes, proteinaceous debris filling airspace and alveolar septal thickening. Each variable received a severity-based score of 0, 1, or 2, with the assigned weights reflecting their individual significance in the evaluation process.

*Flow cytometry analysis*: To analyze the rTEM PMN phenotype of patients, whole blood was isolated from patients with sepsis (sampled less than 48 h after diagnosis) or healthy controls using buffered sodium citrate. Briefly, 100 μL of the blood samples were incubated with anti-CD16/CD32 antibodies (5 mg/mL, 130-092-575, Miltenyi) for 15 min at 4°C to block Fc receptor-mediated antibody binding. Subsequently, the blood samples were stained with the antibodies listed in Table S2 for 30 min at 4°C in the dark after Fc blocking. The samples were then analyzed using an LSRFortessa flow cytometer (BD Biosciences) and FlowJo V10 software (Tree Star, Inc., Ashland, OR, USA).

For analysis of the mouse lung, half of the lung was cut into pieces, and digested with 2.5 mL RPMI 1640 containing collagenase IV (1 mg/mL; C5138; Sigma-Aldrich) and deoxyribonuclease I (50 μg/mL; 10104159001; Roche) in a volume of 2 mL. Following 45 min of incubation at 37°C in a shaker incubator at 125 rpm, the resulting cell suspension was filtered through a 70 μm cell strainer and then treated with erythrocyte lysis buffer (555899; BD) for 5 min. After Fc receptor blocking, single-cell suspensions were then stained with primary antibodies for 30 min at 4°C. The antibodies used were listed in Table S2. Stained cells were analyzed using a flow cytometer (BD LSRFortessaTM, BD Biosciences) and FlowJo V10 software (Tree Star, Inc., Ashland, OR, USA). Background fluorescence levels were determined using Fluorescence Minus One (FMO). After doublet exclusion, live PMN populations were gated as CD45+CD11b+Ly6G+, while the rTEM PMNs required an additional ICAM1+CXCR1- criterion.

A total ROS kit was used to measure ROS level changes in rTEM PMNs according to manufacturer's instructions (88-5930-74, Thermo Fisher Scientific). Cells were loaded with 1× ROS Assay Stain for 60 min in a 37°C incubator, then analyzed at 488 nm in the FITC channel using flow cytometry (BD LSRFortessaTM, BD Biosciences).

For analysis of PMNs apoptosis, an Annexin V-FITC/PI Kit (E-CK-A211, Elabscience) was used according to the manufacturer's protocol. Apoptosis of rTEM PMNs was quantified by the double staining of Annexin V and PI using flow cytometry.

*PMN isolation from mouse bone marrow*: Mouse bone marrow-derived PMNs were isolated as previously described.^[1]^ PMNs were obtained from the bone marrow of C57BL/6 mice by negative selection using PMN isolation kit (130-097-658, Miltenyi) according to the manufacturer’s specifications. The purity of PMNs defined by (CD11b+Ly6G+) was confirmed to be > 95% by flow cytometry analysis (LSRFortessa, BD Biosciences).

*Adoptive cell transfer*: PMNs with the rTEM phenotype were induced *in vitro* by TNF-α (100 U/mL) using an established protocol.^[2]^ The obtained rTEM PMNs (2×10^6/mice) were stained with PE-conjugated anti-Ly6G antibody and then intravenously transferred into wild-type mice following previously described methods. After 3 h, the mice were subjected to analysis using confocal IVM to examine pulmonary PMN retention. Lung tissue samples were collected 12 h after cell transfer for evaluating tissue pathology and inflammation level.

*Wet to dry detection*: As a separate indicator of pulmonary edema formation, Wet to dry (W/D) ratios were determined for both the left and right lungs. The lungs were excised separately and the weight of wet lung was immediately measured. The obtained value was then divided by the corresponding weight after 24 h of drying in a 65°C oven.

*RNA extraction and quantitative real-time PCR assay (qPCR)*: Total RNA was extracted from cells or lung tissues using TRIzol (15596018, Invitrogen), and complementary DNA was synthesized with HiScript II Q RT SuperMix (R223-01, Vazyme). RT-qPCR was performed on the LightCycler96 system (Roche) using ChamQ Universal SYBR qPCR master mix (Q341-02, Vazyme). Gene expression levels were normalized to those of β-actin. The primer sequences used in this study are listed in Table S3.

*Cell culture and treatment*: Mouse pulmonary ECs were cultured in DMEM-F2 (C11765500BT, Gibco) supplemented with 1% antibiotic–antimycotic (15240062, Gibco), 5% fetal bovine serum (FBS; 10099141, Gibco), 1% EC growth supplement (ECGS, 1052, ScienCell), 100 IU/ml heparin (H3149, Sigma-Aldrich), and 92 mg/L d-valine (V1255, Sigma-Aldrich). Exosome-depleted FBS was obtained by ultracentrifugation at 120,000 × g for 16 h. Upon reaching confluence, the cells were gently rinsed twice with PBS (PB180327, Procell), and subsequently treated with lipopolysaccharide (LPS; L2630, Sigma-Aldrich) at a concentration of 1 μg/mL or the vehicle control. This induction was performed using fresh medium supplemented with EV-depleted FBS and allowed to proceed for 24 h.

*EV isolation*: A standard differential centrifugation protocol wase used for EVs isolated from plasma or cell culture supernatants. Whole blood from mice or patients was collected in an ethylenediaminetetraacetic acid (EDTA) coated tube (BD Biosciences, San Jose, CA, USA.) for plasma separation by centrifugation at 1000 × g for 15 min. The cell supernatants collected after culture were first centrifuged at 300 × g for 5 min at 4°C to remove cell debris. Subsequently, the plasma or conditioned media was collected and underwent sequential centrifugation steps: First, at 2000 × g for 20-min at 4°C to further eliminate any residual debris and apoptotic bodies; the resulting supernatants were transferred to new tubes and centrifuged at 13,000 × g for 30 min at 4°C to remove pellet containing large EVs; finally, the supernatants were centrifuged at 200,000 × g for 1.5 h to pellet EVs, which were washed once, and suspended in PBS.

*Characterization of the EVs*: Quantification and size distribution analysis of EVs were performed using NTA (Particle Metrix ZetaView, Meerbusch, Germany). The morphology of the EVs was examined using a TEM (H-7650, Hitachi, Japan). EV-associated markers (CD63, CD9, TSG101, and ALIX), negative markers (Calnexin and GM130), and EC-associated proteins (CD31) were assessed using western blotting. All antibodies were obtained from Abcam (Abcam, UK).

*In vivo treatment*: Each mouse received 10^9 EV particles from different stimulations in 100 μL of PBS via tail vein injection. Control mice received an equal volume of PBS. In the GW4869-mediated EV inhibition experiments, the mice received an intraperitoneal injection of GW4869 (1 mg/kg, D1692, Sigma-Aldrich) once daily for 3 days. EVs interventions were performed 3 days after GW4869 administration. In the GW311616A-mediated NE inhibition experiments, mice were orally pretreated with the NE inhibitor GW311616A (2 mg/kg, HY-15891A, MCE) for 24 h.

*Pulmonary function analysis*: A whole-body plethysmograph system (WBP-4MR, TOW, China) was used to test the pulmonary function in mice because of its non-invasiveness, simplicity, and commercial availability.^[3]^ Mice were placed into the plethysmography chambers for a 30-min acclimatization period. Unrestrained mice were monitored for 10 min after the system was balanced. ^[4]^ The frequency, Penh, peak expiratory flow and tidal volume were determined using the software (ResMass version 1.4.2, TOW, China), as previously described.

*Western blotting analysis*: Protein lysates from EVs, cells, or lung tissues were prepared following standard protocols, and their protein content was quantified using a BCA protein assay kit (P0010, Beyotime Biotechnology). Protein samples were separated on a bis-Tris gel (F11420Gel, ACE) and then transferred onto polyvinylidene difluoride membranes (IPVH00010, Millipore) using a wet transfer system. After blocking with 5% skimmed milk in TBST with Tween 20 (0.1%) for 1 h, the membranes were incubated with primary antibodies overnight at 4°C. The primary antibodies used in this study are listed in Table S4. Subsequently, the membranes were washed and incubated with the appropriate horseradish peroxidase(HPR)-conjugated secondary antibodies for 1 h at room temperature. ECL solution (SQ101; Epizyme Biotech) was then added and the signals were detected using a chemiluminescence imaging system. Intensity values representing relative protein expression were normalized to β-actin.

*Immunofluorescence staining*: Formaldehyde-fixed cells or frozen lung sections were washed three times in PBS and blocked with 5% calf serum in PBS for 1 h at room temperature. Primary and secondary antibodies were diluted in blocking solution. The sections were then washed 3 times in PBS and incubated with the primary antibodies listed in Table S5 overnight at 4°C. After 3 washes in PBS, the sections were incubated with secondary antibodies. Finally, after another 3 times wash in PBS, the sections were stained with DAPI (0100-20; SouthernBiotech) and visualized using a confocal microscope (Leica).

*EV fluorescence labeling*: Purified EVs were fluorescently labeled with Vybrant DiD (V-22887, Invitrogen) following the manufacturer’s instructions. EVs were incubated with DiD (1:1000 dilution in PBS) for 15 min at 37°C. Excess dye was removed by washing the samples in 20 mL of PBS at 200,000 × g for 1.5 h, ultimately yielding DiD-stained EV preparations. DiD-labeled EVs derived from mouse pulmonary ECs were intravenously injected into C57BL/6 mice at a concentration of 10^9 particles per mouse. Tissue samples (lung, spleen, kidney, liver, and heart) were collected at 6, 12, 24, and 48 h after EVs injection for *in vivo* and *ex vivo* imaging.

*Myeloperoxidase (MPO) activity assay*: The MPO activity of lung tissue homogenates were quantified using commercially available enzyme-linked immunosorbent assay (ELISA) kits (ab105136, Abcam) following the manufacturer’s instructions. Lung tissues were homogenized and resuspended in 4 volumes of MPO assay buffer, then centrifuged at 13,000 × g for 10 min to remove any insoluble material. The MPO activity of lung supernatants was measured using a reaction mix including hydrogen peroxide solution II and MPO assay buffer, which subsequently reacts with the DTNB probe to eliminate color in absorbance at 412 nm using a microplate reader (Infinite M200PRO, TECAN). Parallel standard wells were prepared as the background control. Enzyme activity was calculated using a standard curve generated with MPO and expressed as Units/g tissue.

*ELISA*: Protein levels of TNF-α, IL-6, KC, and MIP-2 cytokines in the serum and lung tissue extracts were quantified using commercially available ELISA kits (Raybio Technology) following the manufacturer’s instructions. The following ELISA kits were used: mouse TNF-α (ELM-TNFα), IL-6 (ELM-IL6), KC (ELM-KC), and MIP-2 (ELM-MIP-2).

*Induction of rTEM PMNs in EC cultures by EVs*: A modified approach of a previously documented protocol was employed to generate reverse transmigrated murine PMNs.^[2]^ Briefly, purified PMNs were introduced into the confluent EC monolayer at a concentration yielding six PMNs per EC. Subsequently, EVs (10^9 particles/mL) were added to this system and cultured for 1 h in the cell incubator. Next, non-adherent PMNs were removed by washing and the cells were cultured for an additional 24 h. After 24 h, more than 60% of the initially transmigrated PMNs had back-transmigrated, and were harvested from the apical surface of the ECs by washing the endothelial surface several times with DMEM.

*Cell transfection with KPNB1 shRNA and subsequent EV isolation*: CD63 overexpression fused with GPF lentivirus particles and *KPNB1* short hairpin RNA (shRNA; sh*KPNB1*) lentivirus particles were obtained from Genomeditech Co. Ltd (Shanghai, China). The 3 shRNA sequences are listed in Table S6. The negative control (NC) of these sequences was used. To obtain stably transfected mouse pulmonary ECs, the above lentiviruses were added to cell medium supplemented with polybrene (10 μg/mL, Genomeditech). After transfection for 72 h, the cells were cultured with puromycin (Beyotime Biotechnology) for 7 days. The efficiency of these lentivirus particles was validated and the one with highest efficiency was adopted for subsequent study. For EV isolation, after LPS treatment for 24 h, the cell supernatant of stably transfected ECs or NC stably transfected ECs were collected for further EVs isolation by differential ultracentrifugation.

**References**

[1] Y. Wang, S. Sano, K. Oshima, M. Sano, Y. Watanabe, Y. Katanasaka, Y. Yura, C. Jung, A. Anzai, F. K. Swirski, N. Gokce, K. Walsh, *Circulation* **2019**, *140* (6), 487, https://doi.org/10.1161/circulationaha.118.038820.

[2] A. Woodfin, M. B. Voisin, M. Beyrau, B. Colom, D. Caille, F. M. Diapouli, G. B. Nash, T. Chavakis, S. M. Albelda, G. E. Rainger, P. Meda, B. A. Imhof, S. Nourshargh, *Nat Immunol* **2011**, *12* (8), 761, https://doi.org/10.1038/ni.2062.

[3] a) X. Hu, J. Su, M. Chen, Y. Tu, C. Wu, X. Cao, X. Yuan, F. Zhang, W. Ding, *Sci Total Environ* **2023**, *892*, 164732, https://doi.org/10.1016/j.scitotenv.2023.164732; b) J. Zhou, H. Chen, Q. Wang, S. Chen, R. Wang, Z. Wang, C. Yang, A. Chen, J. Zhao, Z. Zhou, Z. Mao, G. Zuo, D. Miao, J. Jin, *Aging Cell* **2022**, *21* (8), e13680, https://doi.org/10.1111/acel.13680.

[4] a) R. Lim, M. J. Zavou, P. L. Milton, S. T. Chan, J. L. Tan, H. Dickinson, S. V. Murphy, G. Jenkin, E. M. Wallace, *J Vis Exp* **2014**, (90), e51755, https://doi.org/10.3791/51755; b) L. Sun, M. Fan, D. Huang, B. Li, R. Xu, F. Gao, Y. Chen, *Biomaterials* **2021**, *271*, 120761, https://doi.org/10.1016/j.biomaterials.2021.120761.


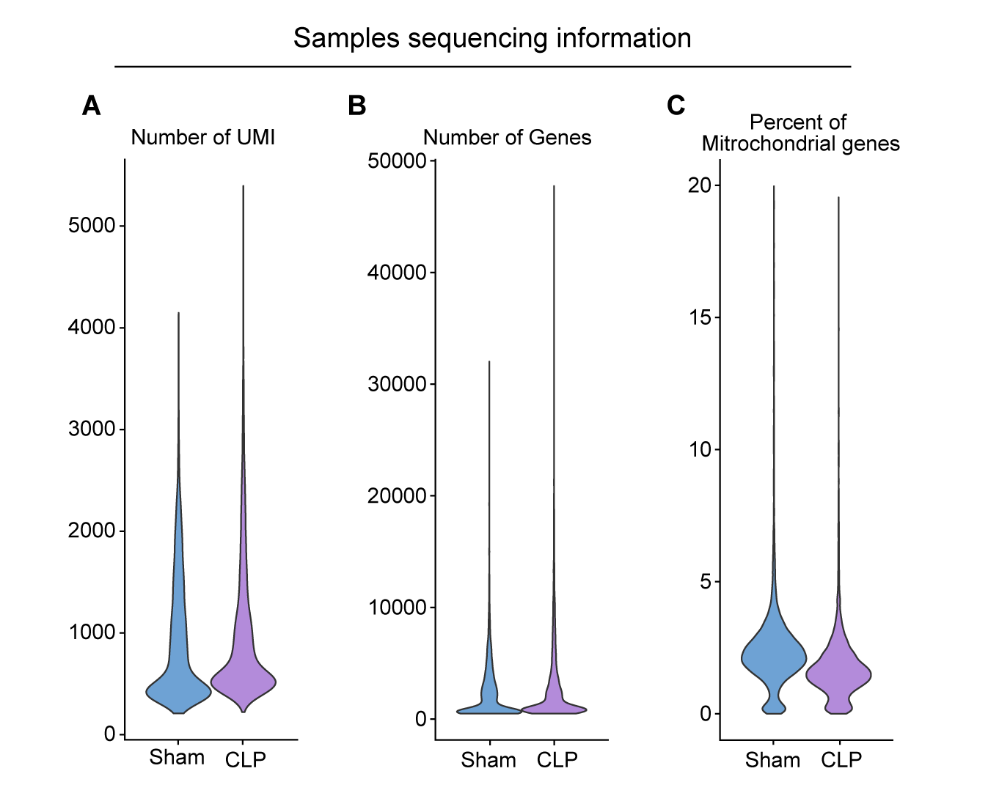


Figure S1. Murine lung scRNA-seq quality control. (A–C) Violin plots depicting the distributions of unique molecular identifier (UMI) counts per cell (A), gene counts per cell (B) and percentage of mitochondrial transcripts per cell (C) from sham (n = 5,329 cells) and CLP (n = 5,650 cells) group.


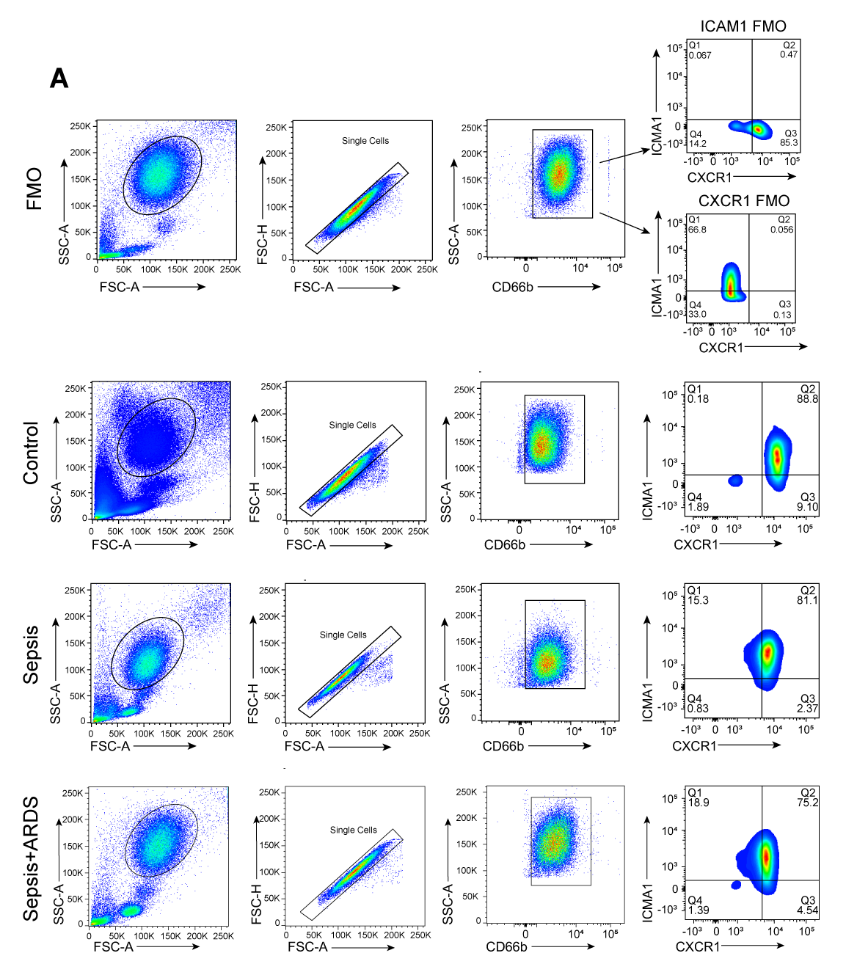


Figure S2. Gating strategy and phenotype for rTEM PMNs in human. (A) Gating strategy for proportion of peripheral rTEM PMNs in healthy controls, patients with sepsis with or without ARDS development related to (Figure 1A).


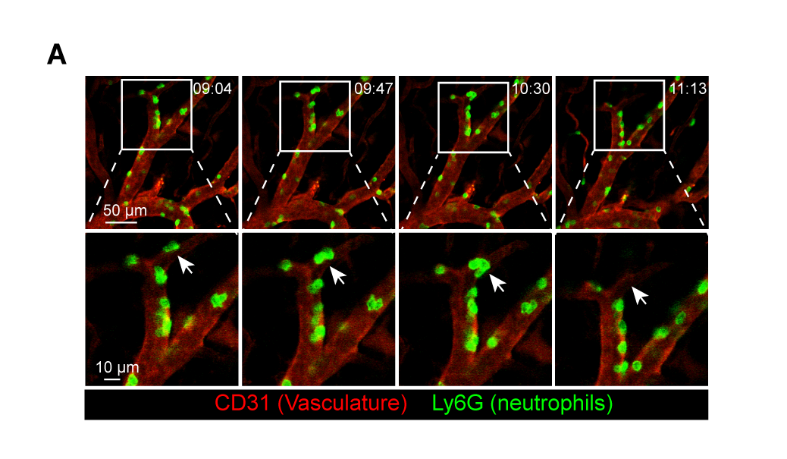


Figure S3. Reverse-transendothelial migration of PMNs in inflamed intestinal vasculature in sepsis. (A) Time-lapse confocal images showing a rTEM event of PMNs labeled by Ly6G (green) in the intestinal vasculature marked by CD31 (red) of a CLP-operated mice with the PMNs (white arrow) in the subendothelial space (t = 09 : 04 min) re-entering the vascular lumen (t = 11: 13 min). Scale bars, 50 µm (wide field) and 10 µm (magnified spot).

**
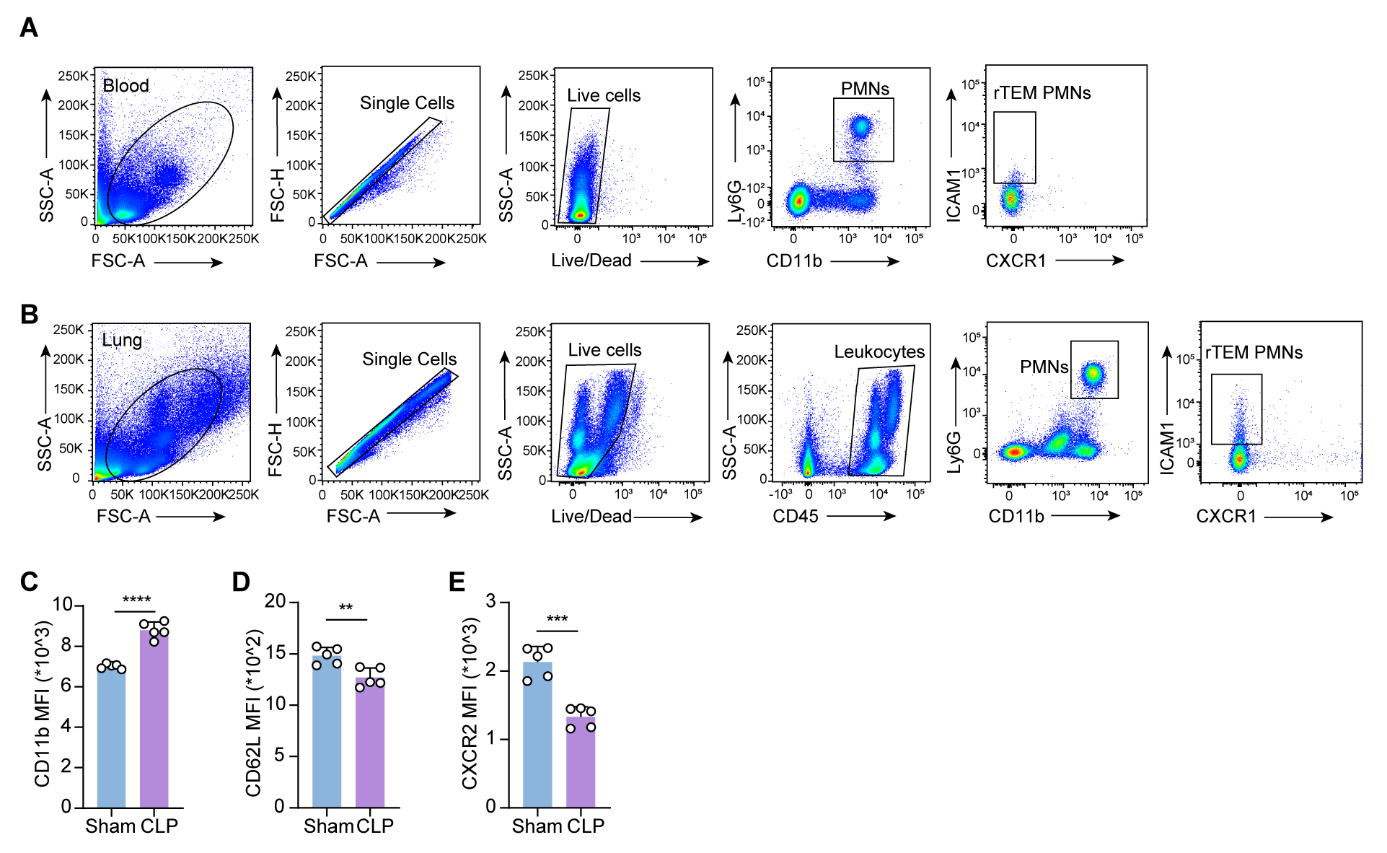
Figure S4.** Gating strategy and phenotype of PMNs and rTEM PMNs in mice. (A, B) Gating strategy for proportions of PMNs and rTEM PMNs in the blood (A) and lungs (B) of sham and CLP-subjected mice euthanized 12 h. (C–E) MFI of CD11b, CD62L, and CXCR2 in pulmonary PMNs of the sham and CLP-operated mice (n = 5). Statistics: unpaired two-tailed *t-test* in (C–E). Data are represented as mean ± SEM. ***P* < 0.01, ****P* < 0.001, and *****P* < 0.0001.


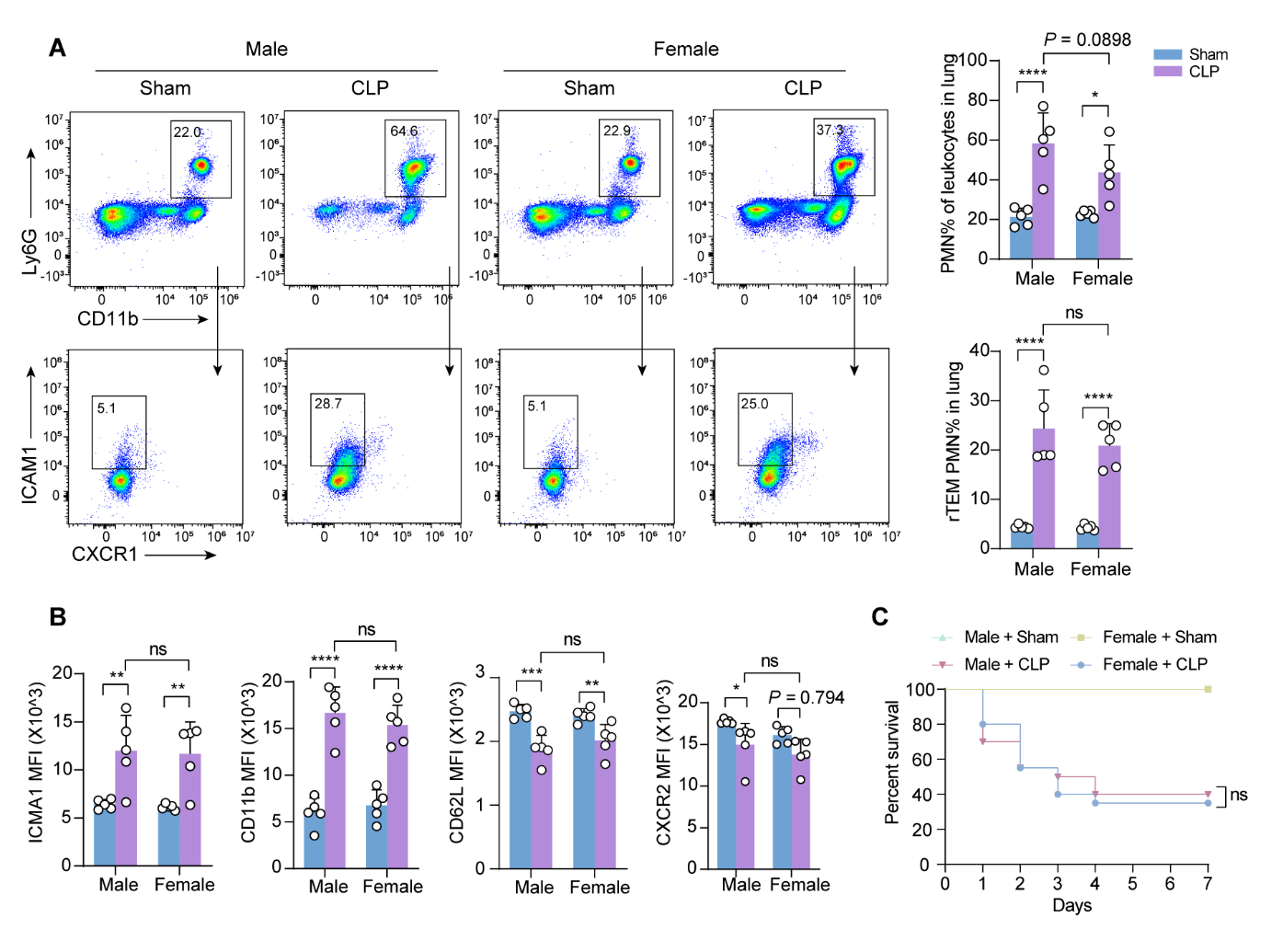


Figure S5. Effect of sex differences on lung PMNs and survival in septic mice. (A) Flow cytometric analysis of PMNs and rTEM PMNs in the lungs of male or female mice 12 h after CLP (n = 5). (B) MFI of ICAM1, CD11b, CD62L, and CXCR2 in the pulmonary PMNs of CLP-operated mice compared to the sham-operated mice (n = 5). (C) Survival rate of mice in each group within 7 days. (n = 8 male or female mice in the sham-operation groups; n = 20 male or female mice in the sham-operation groups. Statistics: two-way ANOVA with Sidak’s multiple comparison test in (A, B); log-rank test (C). Data are represented as mean ± SEM. ns, no significance. **P* < 0.05, ***P* < 0.01, ****P* < 0.001, and *****P <* 0.0001.


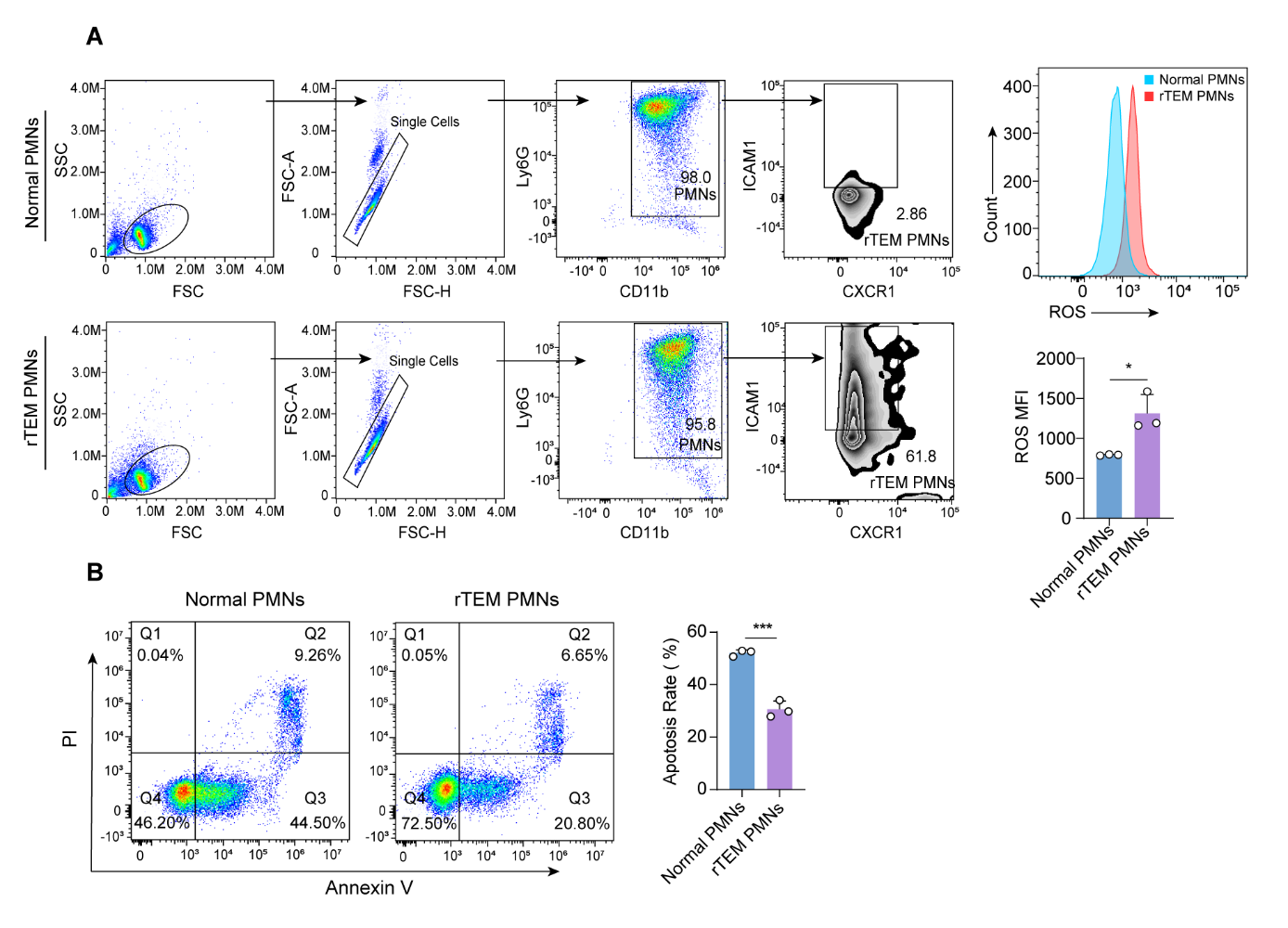


Figure S6. ROS release and apoptosis levels in rTEM PMNs generated *in vitro*. Flow cytometric analysis of the ROS release (A) and apoptosis (B) in rTEM PMNs generated by TNF-α compared to the normal PMNs over 24 h (n = 3). Statistics: unpaired two-tailed *t-test* in (A, B). Data are represented as mean ± SEM. **P* < 0.05, ***P* < 0.01, ****P* < 0.001, and *****P* < 0.0001.

**
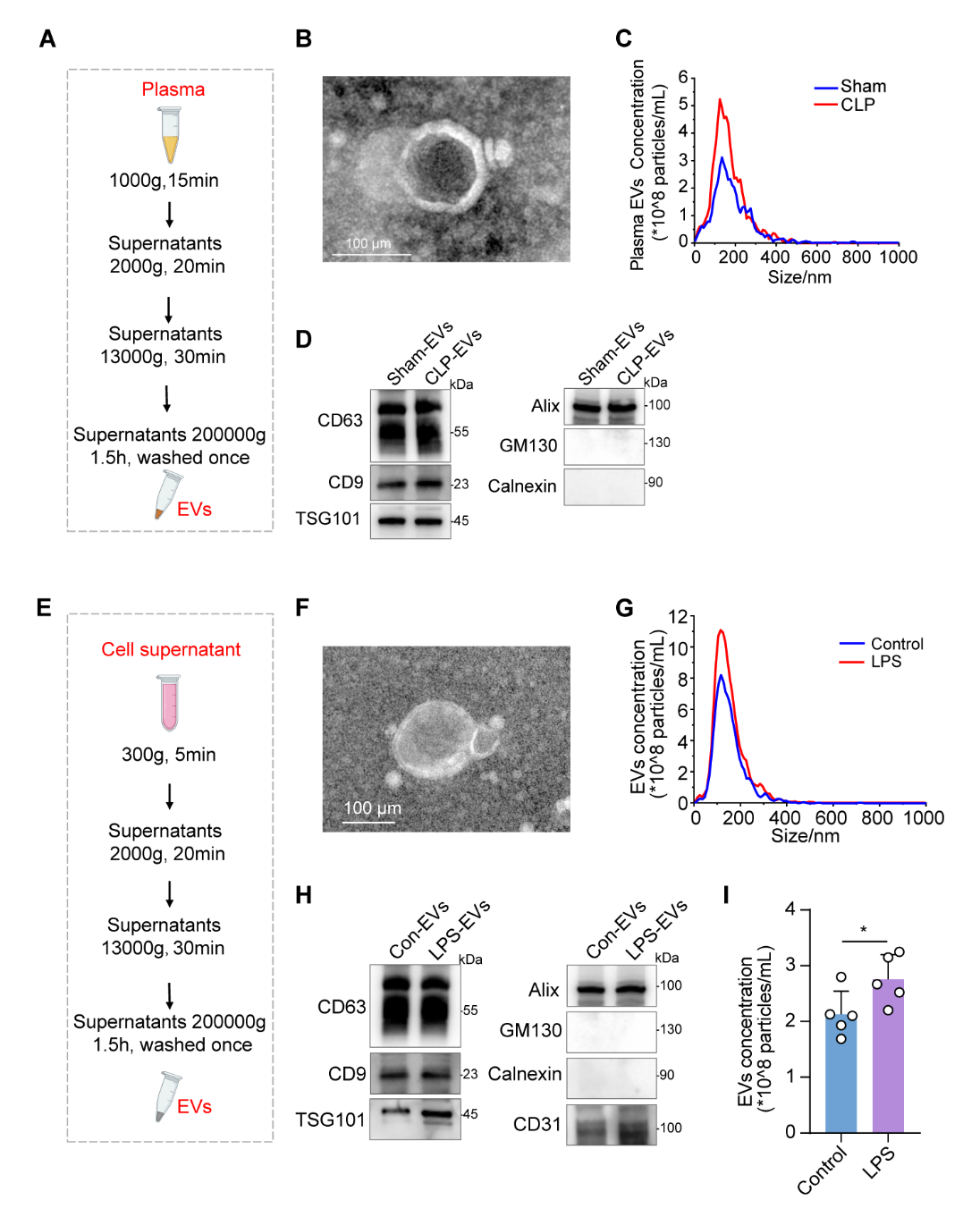
**

**Figure S7.** Characterization of EVs from mouse plasma and cell supernatant. (A) Isolation of plasma EVs by differential centrifugation (Created with BioRender.com). (B) Electron micrograph of uranyl acetate–stained plasma EVs. (C) NTA validation of the size distribution of plasma EVs. (D) Western blotting analysis of EV-associated (CD63, CD9, TSG101 and Alix) and negative markers (GM130 and Calnexin) of plasma EVs. (E) Isolation of EVs from cell supernatant by differential centrifugation (Created with BioRender.com). (F) Electron micrograph of uranyl acetate–stained EC-derived EVs. (G) NTA validation for the size distribution of EVs from ECs. (H) Western blotting analysis of EV-associated markers of EC-derived EVs. (I) Particle concentration of EVs isolated from equal cell supernatant volumes analyzed by NTA (n = 3). Statistics: unpaired two-tailed *t-test* in (I). Data are represented as mean ± SEM. **P* < 0.05.

**
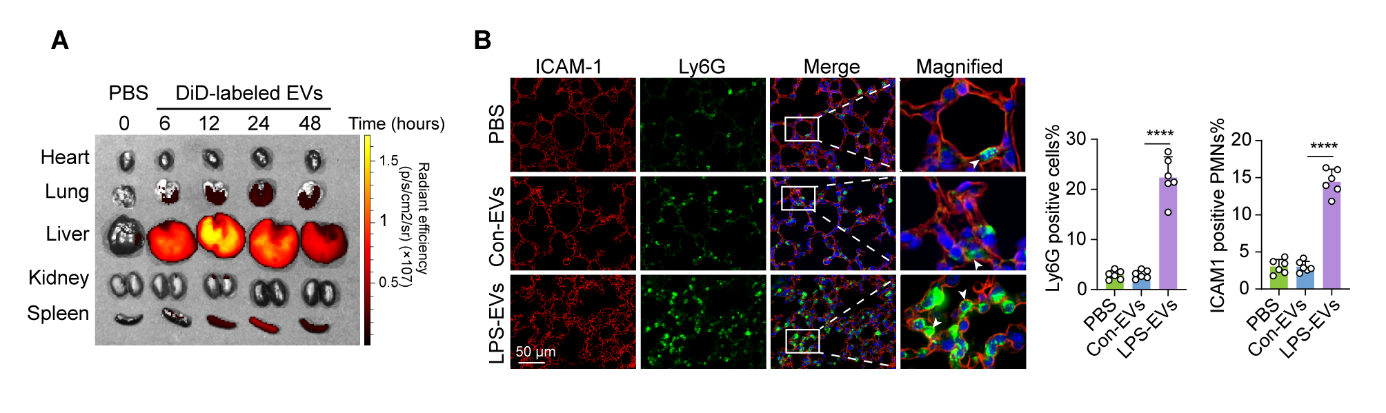
**

Figure S8. *In vivo* biodistribution and pulmonary PMN infiltration after EVs treatment. (A) Imaging of DiD fluorescence intensity of indicated organs at 6, 12, 24, and 48 h after LPS-EVs intravenous injection. (B) Representative immunostaining images for Ly6G (green) and ICAM1 (red). Scale bar, 50 μm. Quantitative analysis of Ly6G positive cells, and ICAM1 positive PMNs in mouse lung samples (n = 5). Statistics: one-way ANOVA with Dunnett’s multiple comparison test in (B). Data are represented as mean ± SEM. *****P* < 0.0001.

**
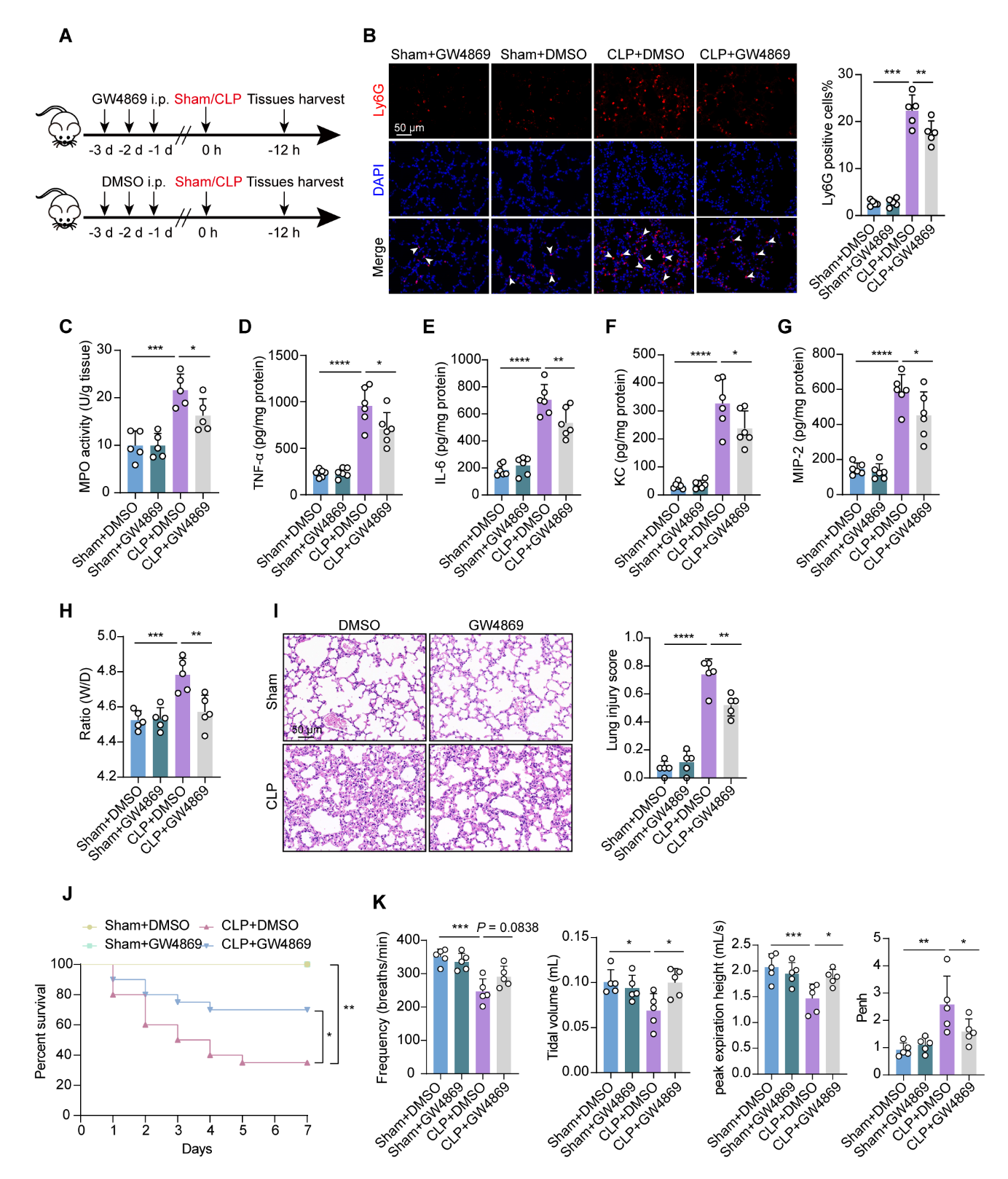
**

Figure S9. Effects of EVs inhibition on pulmonary inflammation and injury in septic mice. (A) Schematic showing GW4869 administration and tissue harvest. (B) Representative images of immunofluorescence staining of Ly6G (red) in the lung sections of mice 12 h after operation. Scale bar, 50 μm. Quantitative analysis of Ly6G positive cells in mouse lung samples (n = 5). (C) MPO activity of lung tissues at 12 h (n = 5). (D–G) Protein expression of TNF-α, IL-6, KC, and MIP-2 in lung tissue homogenates by ELISA (n = 5). (H) W/D ratio of lung tissue at 12 h (n = 5). (I) Representative images of H&E-stained lung sections and lung injury score after GW4869 treatment (n = 5). (J) Survival rate of mice in each group within 7 days. (n = 8 male mice in the sham-treated groups with DMSO or GW4869 treatment; n = 20 male mice in the CLP-treated groups with DMSO or GW4869 treatment). (K) Detection of mice respiratory frequency, tidal volume, peak expiration height, and Penh in different groups by using the whole-body plethysmograph system (n = 5). Scale bar, 50 μm. Statistics: one-way ANOVA with Dunnett’s multiple comparison test in (B–I; K); log-rank test (J). Data are represented as mean ± SEM. **P* < 0.05, ***P* < 0.01, ****P* < 0.001, and *****P* < 0.0001.

**
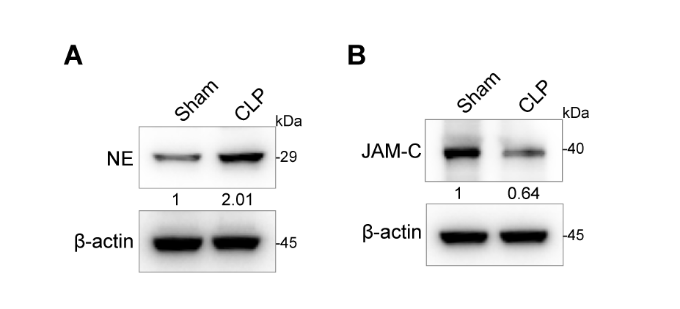
**

Figure S10. Upregulation of NE and cleavage of JAM-C in lung of septic mice. (A and B) Western blotting analysis of NE and JAM-C expression in the lung of mice subjected to CLP at 12 h.


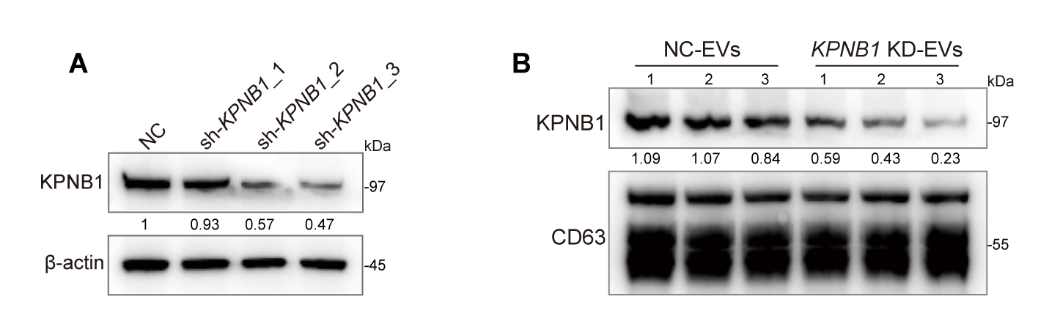


Figure S11. Transfection efficiency in ECs transfected with *KPNB1* shRNA and characterization of *KPNB1* KD-EVs. (A) Western blotting analysis of transfection efficiency in mouse ECs transfected with *KPNB1* shRNA. (B) Western blotting analysis of KPNB1 in EVs obtained from *KPNB1*-knockdown cells or the NC-treated cells stimulated by LPS for 24 h.

Table S1. Clinical characteristics of patients.

| Parameters | Control (n=8) | Sepsis (n=26) | *P value* |
| --- | --- | --- | --- |
| Age (year) | 59.5 (13.5) | 66.0 (13.5) | 0.2321 |
| Gender |  |  | 0.6664 |
| Male | 5 (62.5) | 14 (53.8) |  |
| Female | 3 (37.4) | 12 (46.2) |  |
| Primary site of infection | | | |
| Intra-abdominal | 0 (0) | 18 (69.2) |  |
| Urinary tract | 0 (0) | 6 (23.1) |  |
| Other^b^ | 0 (0) | 2 (7.7) |  |
| SOFA score | 9.6 (4.4) | 1(1.4) | < 0.001 |
| APACHE II score | 18.6(7.5) | 6.75 (5.0) | < 0.001 |
| Sepsis shock | 0 (0) | 19 (73.01) |  |

^b^ Other site of infection except intrapulmonary infection.

Categorical variables are expressed as n (%), and continuous variables are expressed as the mean (SD).

Abbreviations: SOFA, Sequential Organ Failure Assessment; APACHE, Acute Physiology and Chronic Health Evaluation.

Table S2. Antibodies used for flow cytometry

| **Antibody** | **Company** | **Catalog #** | **Dilute** |
| --- | --- | --- | --- |
| APC conjugated Anti-human CD66b | Biolegend | 305125 | 1:100 |
| PE conjugated Anti-human ICAM1 | Biolegend | 353106 | 1:100 |
| BV605 conjugated Anti-human CXCR1 | BD | 743421 | 1:100 |
| APC-Cy7 conjugated Anti-mouse CD45 | BD | 557659 | 1:100 |
| FITC conjugated Anti-mouse CD11b | BD | 557396 | 1:100 |
| APC conjugated Anti-mouse Ly6G | BD | 560599 | 1:100 |
| BV786 conjugated Anti-mouse ICAM1 | BD | 740844 | 1:100 |
| PE conjugated Anti-mouse CXCR1 | BD | 566383 | 1:100 |
| BV421 conjugated Anti-mouse CXCR2 | BD | 566622 | 1:100 |
| BV650 conjugated Anti-mouse CXCR4 | BD | 740523 | 1:100 |
| PerCP-Cy5.5 conjugated Anti-mouse CD62L | BD | 560513 | 1:100 |
| Viability Dye eFluor 510 | BD | 564406 | 1:100 |
| FITC-conjugated anti-mouse CD31 | BD | 558738 | 1:100 |

Table S3. Sequences of primers for qPCR of mouse lungs.

| Gene Sequence (5’ to 3’) |
| --- |
| mouse-Tnf-α-F CGAGTGACAAGCCTGTAGCC  mouse-Tnf-α-R AAGAGAACCTGGGAGTAGACAAG |
| mouse-*Il-6*-F ACCAGAGGAAATTTTCAATAGGC  mouse-*Il-6*-R TGATGCACTTGCAGAAAACA |
| mouse-*Il-1β*-F GGTCAAAGGTTTGGAAGCAG  mouse-*Il-1β*-R TGTGAAATGCCACCTTTTGA |
| mouse-*Kc*-F TGAGCTGCGCTGTCAGTGCCT  mouse-*Kc*-R AGAAGCCAGCGTTCACCAGA |
| mouse-*Mip-2*-F CCAACCACCAGGCTACAG-  mouse-*Mip-2*-R CTTCAGGGTCAAGGCAAAC |
| mouse-*β-actin*-F GTGACGTTGACATCCGTAAAGA  mouse-*β-actin*-R GCCGGACTCATCGTACTCC |

**Table S4. Antibodies used for western blotting.**

| **Antibody** | **Company** | **Catalog #** | **Dilute** |
| --- | --- | --- | --- |
| Rabbit anti-CD31 | Abcam | ab217345 | 1:1000 |
| Rabbit anti-CD9 | Abcam | ab92726 | 1:1000 |
| Rabbit anti-TSG101 | Abcam | ab125011 | 1:5000 |
| Rabbit anti-ALIX | Abcam | ab186429 | 1:1000 |
| Rabbit anti-Calnexin | Abcam | ab52649 | 1:1000 |
| Rabbit anti-GM130 | Abcam | ab52649 | 1:1000 |
| Rabbit anti-CD31 | Abcam | ab222783 | 1:2000 |
| Goat anti-JAM-C | R&D | AF1213 | 0.1 μg/mL |
| Rabbit anti-NE | Affinity | AF0010 | 1:1000 |
| Rabbit anti-KPNB1 | Proteintech | 10077-1-AP | 1:1000 |
| Rabbit anti-STAT1 | CST | 14994T | 1:1000 |
| Rabbit anti-phospho-STAT1 | CST | 9167T | 1:1000 |
| Anti-rabbit IgG HRP-linked | Beyotime | A0208 | 1:2500 |
| Anti-goat gG HRP-linked | Epizyme | SQ101 | 1:5000 |
| Rabbit anti-β-actin | CST | 4973S | 1:1000 |

Table S5. Antibodies used for immunofluorescence.

| **Antibody** | **Company** | **Catalog #** | **Dilute** |
| --- | --- | --- | --- |
| rabbit anti-MPO | Abcam | ab208670 | 1:200 |
| rat anti-ICMA1 | Abcam | ab222736 | 1:200 |
| rat anti-Ly6G | BD | 551459 | 1:200 |
| Goat anti-JAM-C | R&D | AF1213 | 5 μg/mL |
| Rabbit anti-NE | Affinity | AF0010 | 1:200 |

Table S6. shRNA sequence used in this study.

| NO. | TargetSeq |
| --- | --- |
| NC | TTCTCCGAACGTGTCACGT |
| sh*KPNB1*-1 | CCGGAGCACACCAGCAAATTT |
| sh*KPNB1*-2 | CAATGATCCATGAACTATTAA |
| sh*KPNB1*-3 | GACATCGAAAGATCCAGATAT |

Movie S1.

Real-time intravital imaging of PMNs in the pulmonary microcirculation of mice subjected to sham surgery.

Movie S2.

Real-time intravital imaging of PMNs in the pulmonary microcirculation of mice subjected to CLP.

Movie S3.

Real-time intravital imaging of PMNs undergoing rTEM in the pulmonary microcirculation of mice subjected to CLP.

Movie S4.

Real-time intravital imaging of PMNs in the pulmonary microcirculation of mice following intravenous adoptive transfer of PE-labeled normal PMNs.

Movie S5.

Real-time intravital imaging of PMNs in the pulmonary microcirculation of mice following intravenous adoptive transfer of PE-labeled rTEM PMNs.

Movie S6.

Real-time intravital imaging of PMNs in the pulmonary microcirculation of mice intravenously injected with DiD-labeled Con-EVs.

Movie S7.

Real-time intravital imaging of PMNs in the pulmonary microcirculation of mice intravenously injected with DiD-labeled LPS-EVs.
